# Site-specific time heterogeneity of the substitution process and its impact on phylogenetic inference

**DOI:** 10.1186/1471-2148-11-17

**Published:** 2011-01-14

**Authors:** Béatrice Roure, Hervé Philippe

**Affiliations:** 1 Département de Biochimie, Centre Robert-Cedergren, Université de Montréal, Succursale Centre-Ville, Montréal (Québec) H3C 3J7, Canada

## Abstract

**Background:**

Model violations constitute the major limitation in inferring accurate phylogenies. Characterizing properties of the data that are not being correctly handled by current models is therefore of prime importance. One of the properties of protein evolution is the variation of the relative rate of substitutions across sites and over time, the latter is the phenomenon called heterotachy. Its effect on phylogenetic inference has recently obtained considerable attention, which led to the development of new models of sequence evolution. However, thus far focus has been on the quantitative heterogeneity of the evolutionary process, thereby overlooking more qualitative variations.

**Results:**

We studied the importance of variation of the site-specific amino-acid substitution process over time and its possible impact on phylogenetic inference. We used the CAT model to define an infinite mixture of substitution processes characterized by equilibrium frequencies over the twenty amino acids, a useful proxy for qualitatively estimating the evolutionary process. Using two large datasets, we show that qualitative changes in site-specific substitution properties over time occurred significantly. To test whether this unaccounted qualitative variation can lead to an erroneous phylogenetic tree, we analyzed a concatenation of mitochondrial proteins in which Cnidaria and Porifera were erroneously grouped. The progressive removal of the sites with the most heterogeneous CAT profiles across clades led to the recovery of the monophyly of Eumetazoa (Cnidaria+Bilateria), suggesting that this heterogeneity can negatively influence phylogenetic inference.

**Conclusion:**

The time-heterogeneity of the amino-acid replacement process is therefore an important evolutionary aspect that should be incorporated in future models of sequence change.

## Background

With the expansion of genome projects, phylogenomics - the use of numerous genes to infer phylogenetic trees - is becoming a common way to resolve controversial relationships (e.g. [1-5]). Since large datasets increase the amount of phylogenetic signal included in the analysis, phylogenomics is less subject to stochastic errors than single gene phylogenies. Nevertheless, some nodes remain unresolved even at the genome-scale level [6-9]. This can either be due to intrinsic properties of the data, (i.e., short internal branches due to speciation events closely spaced in time) or to inadequate inference methods [10]. In fact, systematic errors may be more pronounced in phylogenomics: in some cases, the gain in phylogenetic signal is masked by an increased level of systematic error, which can attain the same order of magnitude [8]. In the worst case, this leads to erroneous phylogenies with a high statistical support [11,12]. Molecular sequence evolution exhibits a high complexity that is not fully accounted for in current models of sequence evolution. Since the first substitution model [13] several simplifying assumptions have been relaxed; the evolutionary process is considered as heterogeneous (i) between character states, (ii) over time and (iii) along the alignment. Models that relaxed these assumptions (e.g. empirical exchangeability matrices [14], non-stationary nucleotide content [15], or gamma distribution of rates across sites [16]) improve fit to the data and phylogenetic accuracy, and are therefore widely used.

More recently, probabilistic models have been developed to take into account heterogeneity of the qualitative aspect of amino-acid replacements along the alignment, by assigning sites to different classes of substitutional processes [17-25]. Of particular interest is the CAT model [24], a mixture model that infers categories from the data without any *a priori *biological assumptions and takes advantage of the Dirichlet process prior [26] to control the number of categories through a set of hyperparameters (i.e. an infinite mixture model). In this model, the substitution process is assumed to be site-independent and is entirely defined by the equilibrium frequencies of amino acids, while their exchangeabilities are assumed equal (i.e. Poisson process). The equilibrium frequencies over the twenty amino acids constitute a good proxy to represent the functional constraints acting on each position during evolution. In the following, we will call such categories *substitution profiles*, or simply *profiles*. The number of profiles is often several hundreds, showing that previous models lack flexibility in handling heterogeneity of the substitution process across sites and, for large datasets, the CAT model has a better fit to the data than standard models based on substitution matrices (e.g. JTT, WAG or GTR) [5,24,27-30] and renders phylogenetic inference less sensitive to long branch attraction artefact [5,10,31-34]. Despite that the inferred number of categories is large, it has been shown, using a posterior predictive approach, that the number of categories estimated by the CAT model is conservative [24].

The models that assume different evolutionary processes across sites consider that they are homogeneous over time. However, except for a few constant positions for which the evolutionary constraints are uniformly strong, the functional constraints of most positions are likely to have changed over their evolutionary history [35,36]. Indeed, some amino acids are replaced at sites without a significant effect on function, but these changes might modify the environment of the protein, the intra- or inter-protein interactions, and so on, leading to changes of the selective pressure at other sites [35]. This results in variation of the site-specific evolutionary rate across time [35,36], a phenomenon called heterotachy [37], which is recurrent in biological sequences [38-40]. To handle this heterogeneity, models have been proposed that allow the substitution rate to vary over time [41-46].

In this study, we will extend to the principle of heterotachy to the heterogeneity of the substitution process over time. We call homopecilly (ποικιλλω, pecilly, means to vary in Greek) the hypothesis of an identical substitution process over time at a given site. Since functional constraints acting on proteins change over time, we however expect that not only the rate but also the amino acid substitution process may vary. In particular, the subset of acceptable amino acids or the exchangeability matrix at a given site may change throughout evolutionary time. We will therefore test the hypothesis of homopecilly by evaluating whether the nature of the substitution process varies significantly over time at a given site. Briefly, the substitution process will be characterized by the set of stationary frequencies of amino acids, as estimated by the CAT model [24]. Several large datasets will be divided into monophyletic taxa to test the null hypothesis of a homogeneous substitution process, that is a site should be affiliated to the same CAT category (i.e. a set of stationary frequencies) in all predefined monophyletic groups. We demonstrate not only that this null hypothesis is significantly rejected, but also that heteropecilly might generate phylogenetic artifacts.

## Results and Discussion

### Evidence for a significant qualitative heterogeneity over time

**Figure 1:**
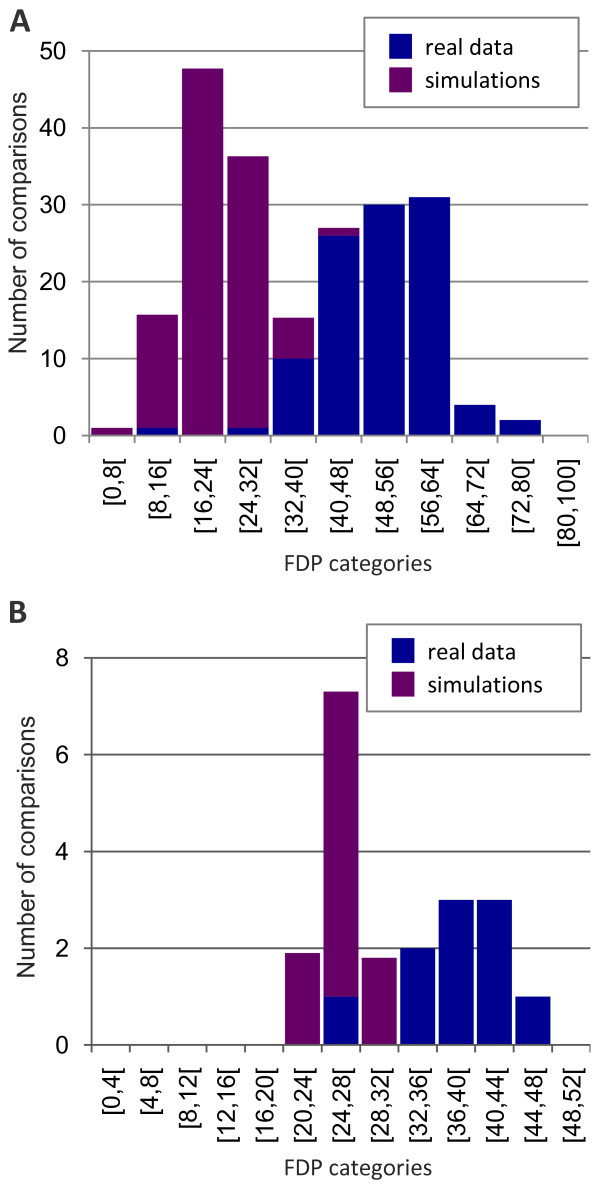
**Stack distributions of FDP**. Values are drawn in blue and purple for real and simulated data, respectively. Histograms are plotted for the 105 pairwise comparisons from the mt336 dataset (A) and the 10 pairwise comparisons from the nuc80 dataset (B). Simulation values were averaged over ten simulated datasets and only variable positions (i.e. two or more substitutions per site), which have sufficient phylogenetic signal for profile affiliation, were considered.

To globally estimate the presence of heteropecilly, the Frequency of Different Profiles (FDP), the frequency of positions that are stably affiliated to two different profiles in a pair of taxonomic groups, is computed (Figure 1). For each comparison (105 and 10 pairwise comparisons for the mt336 and the nuc80 dataset, respectively), only positions showing at least two substitutions are considered, as very slowly evolving positions do not contain a signal strong enough to provide stable affiliations (data not shown). For both datasets, most of the comparisons show high values of FDP: between 40% and 80% for the mitochondrial dataset (Figure 1A), and between 24% and 48% for the nuc80 dataset (Figure 1B). In other words, about half of the stably affiliated positions are best described by two different profiles in two different taxonomic groups. Importantly, the distribution of FDPs is clearly shifted to lower values in simulations under homopecilly than in real data, demonstrating that the observed heteropecilly is not due to stochastic variations.

**Figure 2:**
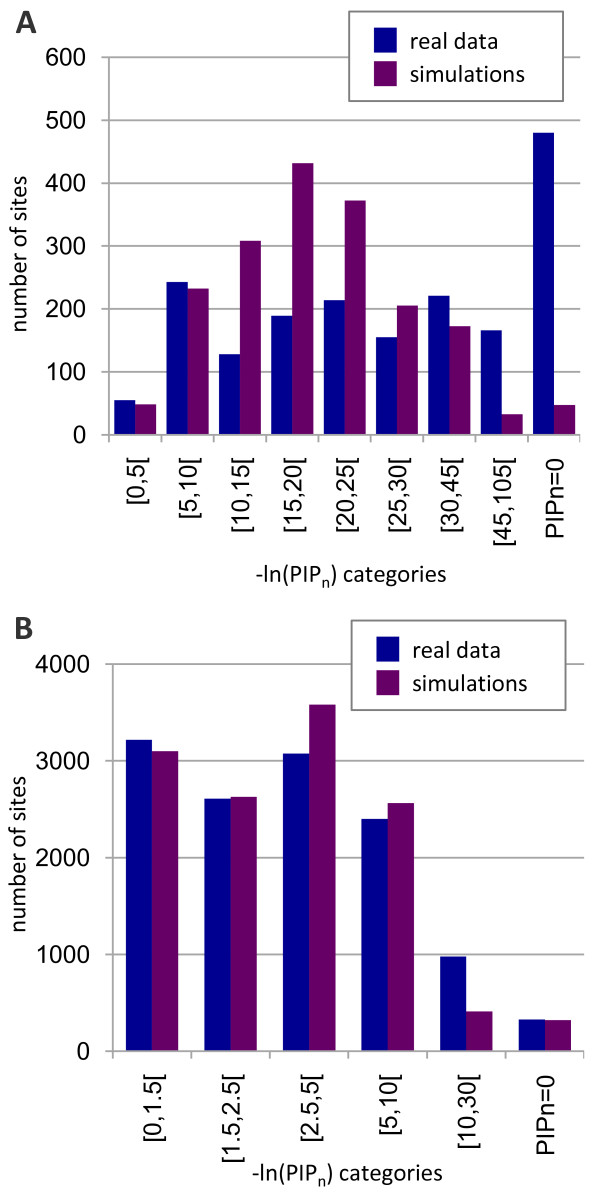
**Distribution of PIP_n_**. Values are drawn for real and simulated data (average of 10 simulated datasets) based on -ln(PIP_n_) categories, in blue and purple for real and simulated data, respectively. Histograms are for the mt336 dataset (A) and the nuc80 dataset (B)

The significance of the FDP statistics is limited by the fact that only 15-49% of the sites are considered. To increase the number of sites stably affiliated, it would be necessary to increase the number of substitutions, hence the number of species in predefined monophyletic groups. However, if heteropecilly is frequent, this will have the effect of increasing the probability that a site changed evolutionary properties, and hence cannot be stably affiliated to a single profile. To eschew this dilemma, we devised another criterion, the Probability of Identical Profile over n clades (PIP_n_), to estimate the heteropecilly for all sites (see Material and Methods). A PIP_n _value of 0 indicates that the site is described by different CAT profiles in at least two groups, whereas a value close to 1 indicates that the site is always affiliated to the same profiles. The distribution of -ln(PIP_n_) for real and simulated datasets is displayed in figure 2. As expected, we observe an excess of sites with high values of -ln(PIP_n_) in real data, whereas the number of sites that show a medium value of -ln(PIP_n_) is higher for the simulated data; results are highly significant (Kolmogorov-Smirnov test, p < 2.2e^-16 ^for both datasets). Moreover, since the PIP_n _criterion is highly correlated with evolutionary rate (see below), the lower evolutionary rate in nuclear genes, relative to mitochondrial genes, decreases the power of the PIP_n _test, making heteropecilly less marked in Figure 2B than in Figure 2A.

Altogether, both the FDP that considers all stably affiliated sites in a pairwise comparison and the PIP_n _that considers every site separately, while comparing all taxonomic groups simultaneously, demonstrate that qualitative time-heterogeneity of the substitution process-which we refer to as heteropecilly-is widespread in real data. Heteropecilly might be due to the comparison of paralogous genes, with different functions. However, the genes encoded in the mitochondrial genome of animals have an extremely high probability of being truly orthologous, since no protein encoding gene duplication is known in these organelles. Similarly, the orthology was carefully checked in the nuclear supermatrix using the protocol described in [5]; in fact, the proteins considered, mainly ribosomal proteins, are not subject to frequent gene duplications in animals and more importantly the duplicates do not change its function. As a result, it is unlikely that the observed heteropecilly is due to the comparison of paralogous genes.

### Relationship between heteropecilly and other biological properties

We first evaluated whether heteropecilly is related to the evolutionary rate (Tables 1 and 2, and Additional file 1: Figures S7A and S7D). Sites with a PIP_n _equal to 0 are generally fast-, or even very fast-, evolving. For instance, more than five-sixths of such nuclear positions have accumulated over 20 substitutions, whereas only 1.5% have undergone less than 9 substitutions. The relationship between heteropecilly and evolutionary rate is highly significant (χ^2 ^test of homogeneity rejected at p = 0 and 4 × 10^-138 ^for nuc80 and mt336 datasets, respectively). This result is expected. A slowly evolving position is under strong functional pressure that limits not only the number of substitutions, but also the diversity of acceptable amino acids, thus reducing the probability of being affiliated to different profiles in different clades. Moreover, the statistical power is reduced. In contrast, a fast-evolving position has more opportunities to explore different types of selective constraints, and therefore accepts substitutions in different ways over time; this leads to a small value of PIP_n_. It is important to note that Tables 1 and 2 suggest that many fast evolving positions are indeed under rather strong selective pressure. If they were completely free to vary, these fast evolving sites would explore all the twenty amino acids. In such cases, substitution profiles would be the same in different clades, characterized by a small equilibrium frequency for many amino acids, and these sites would be associated with a PIP_n _far from 0. In contrast, a small PIP_n _indicates that negative selection in a given clade is strong (i.e. with only a few acceptable amino acids), but that this selection pattern changes over time; for instance, a position might accept only Asp and Glu in one clade, and only Asp and Asn in another.

**Table 1:** Distribution of sites according to the substitution number and -ln(PIPn) for the nuc80 dataset

substitution number	-ln(PIPn)				
	[0,1.5[	[1.5,2.7[	[2.7,5.5[	[5.5,30[	PIPn = 0
[0,3.5[	1288	1218	505	234	0
[3.5,9[	872	813	926	559	5
[9,20[	626	593	907	988	53
[20,80[	432	384	690	1246	269

**Table 2:** Distribution of sites according to the substitution number and -ln(PIPn) for the mt336 dataset

substitution number	-ln(PIPn)				
	[0,12[	[12,22[	[22,33[	[33,105[	PIPn = 0
[0,9[	150	160	115	41	4
[9,26[	107	95	115	100	43
[26,56[	60	73	83	113	132
[56,210[	24	37	21	77	301

A relationship between heteropecilly and rate heterogeneity over time (heterotachy) is possible, since both heterogeneities are due to changes in functional constraints. Accordingly, Tables 3 and 4 (and Additional file 1: Figure S7B and S7E) reveal a link of qualitative heterogeneity with heterotachy (χ^2 ^test of homogeneity rejected at p = 2 × 10^-6 ^and 2 × 10^-18 ^for nuclear and mitochondrial datasets, respectively). For instance, 80% of mitochondrial positions with a PIP_n _of 0 are very heterotachous (p < 1%), whereas only 65% of all positions are heterotachous at that level. Nevertheless, heterotachy and heteropecilly appear anti-correlated: the proportion of heteropecillous sites is greater when sites are less heterotachous for the nuclear dataset, whereas proportion of heteropecillous sites increases with heterotachy for the mitochondrial dataset. Since the species number (hence the number of substitutions) is reduced and some missing data are present in the nuclear alignment, estimation of heterotachy and of heteropecilly is less accurate than in the mitochondrial alignment. More importantly, the relationship with heterotachy is not only less marked than with the evolutionary rate, but it is in fact mainly a consequence of the correlation between heterotachy and evolutionary rate (χ^2 ^test of homogeneity rejected at p = 1 × 10^-7 ^and 1 × 10^-60 ^for nuclear and mitochondrial datasets, respectively): when only the fast evolving positions (i.e. with more than 20 substitutions) are considered, heteropecilly is almost unrelated to heterotachy, especially for the mitochondrial dataset (p = 0.007 and 0.13 for nuclear and mitochondrial datasets, respectively). Further studies are therefore needed to determine whether a change of rate over time is independent, or not, of the fact that a site may be affiliated to different amino acid profiles over time. The absence of a strong correlation between heteropecilly and heterotachy makes sense because the two heterogeneities do not apply on the same criteria: heterotachy is mainly due to loss or gain of functional constraints (i.e. variable strength of constraints), whereas heteropecilly is rather due to variation in the nature of functional constraints, not in their strength.

**Table 3:** Distribution of sites according to the p-value of the heterotachy test and -ln(PIPn) for the nuc80 dataset

	-ln(PIPn)				
heterotachy	[0,1.8[	[1.8,3.45[	[3.45,6.6[	[6.6,30[	PIPn = 0
[0,0.01]	68	51	89	108	4
]0.01,0.05]	165	156	196	206	23
]0.05,100]	1895	1919	1832	1820	299

**Table 4:** Distribution of sites according to the p-value of the heterotachy test and -ln(PIPn) for the mt336 dataset

	-ln(PIPn)				
heterotachy	[0,12[	[12,22[	[22,33[	[33,105[	PIPn = 0
[0,0.01]	152	149	146	241	389
]0.01,0.05]	48	38	46	37	56
]0.05,100]	68	87	82	37	34

We analyzed the correlation between heteropecilly and change in hydropathy, using the standard deviation of the site-wise Profile Hydrophobic Score (PHS) index, which measures the diversity of hydropathy a site displays in various clades. Tables 5 and 6 (see also Additional file 1: Figures S7C and S7F) demonstrate a highly significant relationship (χ^2 ^test of homogeneity rejected at p = 3 × 10^-262 ^and 9 × 10^-189 ^for nuc80 and mt336 datasets, respectively). If we make the assumption that a change in substitution profile reflects a variation in functional constraints, the correlation between the PIP_n _criterion and the PHS score suggests that, when functional constraints change over time, the new spectrum of acceptable amino acids becomes biochemically different from the previous one. Nevertheless, changes from one profile to another are much easier to detect with our protocol when no acceptable amino acids are common to both than when at least one amino acid remains in the set of acceptable residues. In the first case, sites will surely be affiliated to different profiles in different clades, even if the signal is weak, whereas in the second case the sites will be likely affiliated to several, overlapping, profiles showing closer hydrophobic scores. The use of larger datasets (in terms of species number) is required to address this issue.

**Table 5:** Distribution of sites according to the standard deviation of the PHS score and -ln(PIPn) for the nuc80 dataset

	-ln(PIPn)				
|SD(PHS)|	[0,1.5[	[1.5,2.7[	[2.7,5.5[	[5.5,30[	PIPn = 0
[0,0.1[	1676	385	247	83	1
[0.1,0.25[	1895	730	768	157	5
[0.25,0.75[	567	756	1291	938	28
[0.75,5[	37	180	722	1849	293

**Table 6:** Distribution of sites according to the standard deviation of the PHS score and -ln(PIPn) for the mt336 dataset

	-ln(PIPn)				
|SD(PHS)|	[0,12[	[12,22[	[22,33[	**[33,105**[	PIPn = 0
[0,0.33[	231	104	89	26	8
[0.33,0.57[	80	148	132	62	36
[0.57,1[	29	94	87	121	132
[1,2.6[	1	19	26	122	304

Finally, we estimated whether changes between profiles showing similar physico-chemical properties (distributed among these five groups: small, aliphatic, aromatic, charged, other; see Materials and Methods) are more frequent than changes between profiles with different properties. Only sites that are stably affiliated in two different clades are considered. About half of sites (44% and 48%, for mt336 and nuc80, respectively) are affiliated to profiles with different biochemical properties. This suggests that heteropecilly is driven not only by different fine-grained functional constraints, but also by important functional changes. Analyses at the codon level [47] are nevertheless required to estimate whether heteropecilly is driven by positive selection or is the result of changes in purifying selection properties.

### Phylogenetic structure of heteropecilly

**Figure 3:**
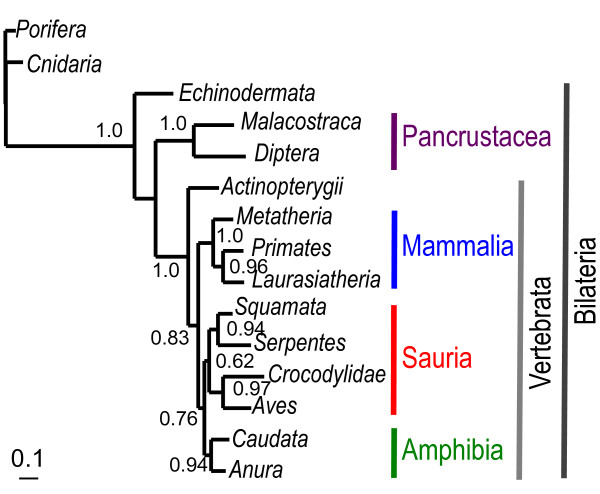
**Topology inferred with a GTR+Γ_4 _model from the mt336 dataset recoded using stably affiliated profiles**. The posterior probabilities, greater than 0.5, are plot on the nodes.

We have shown that the substitution process, as characterized by the CAT profiles, varies over time. Although the way profile affiliations change over time is not known, it is reasonable to assume that profile affiliations are often inherited from ancestors. In other words, for orthologs, two sister clades would generally have the same substitution process, i.e. a given site would generally be affiliated to the same profile in two sister clades. Therefore, a phylogenetic signal is expected to exist in the profile distribution. We tested this hypothesis by recoding the multiple amino acid sequences of a clade into a single artificial sequence. In these new sequences, the state of a given site is the profile to which this site is stably affiliated or a question mark otherwise. Only the 20 most frequent profiles are considered (see Materials and Methods for details) and trees are inferred using the GTR+Γ_4 _(Figure 3) and the CAT+Γ_4 _(Additional file 1: Figure S9) models.

With the mt336 dataset, most of the known clades are recovered with the GTR+Γ_4 _model (Figure 3), many of them with high posterior probabilities (PP), e.g. Bilateria, Pancrustacea, Vertebrata, Amphibia, Sauria, Archosauria, Lepidosauria, Theria and Eutheria. The only problematic case is the relative position of Amphibia, Mammalia and Sauria. The mono/paraphyly of Deuterostomia is known to be a difficult question, even using a hundred nuclear genes [27]. Although we don't know which statistical model has to be used for analyzing the artificial recoded sequences, recovering at least some clades is reassuring. To test the possibility of a correct grouping of clades by chance, we applied the recoding protocol to the ten datasets simulated under a homopecillous model (sites should therefore be affiliated to the same profile whatever the clade considered, except because of stochastic error which should not be phylogenetically structured): except in one case, the expected monophyletic groups are not recovered or only recovered with very small posterior probabilities (Additional file 2: Table S7). Similar results were obtained for the nuc80 dataset (data not shown), but are less clear because only five clades are available.

The congruence of the phylogeny inferred from recoded data with current taxonomy demonstrates the presence of a strong phylogenetic signal in changes of the evolutionary process, especially since only few parsimony informative positions are available (353 and 120 for the recoded mitochondrial and nuclear datasets, respectively). Changes in the evolutionary process are therefore relatively rare since sites remain affiliated to the same profile over hundred millions of years. The same observation has been made for evolutionary rates, leading to local molecular clock [48] or auto-correlated relaxed molecular clock models [49]. Nevertheless, the incongruence observed in a few cases indicates that homoplasy is present in recoded data and is not correctly handled by the CAT+Γ model; not surprisingly, when a profile affiliation changes, it can either revert to the ancestral state or converge toward a state independently acquired in a distantly related clade. The misleading effect of homoplasy is enhanced by the very heterogeneous rate of change of profile affiliations: for the mitochondrial dataset, the rate is high on the branch leading to Bilateria (Figure 3) and for the nuc80 dataset, nematodes and platyhelminthes evolved several times faster than the other bilaterians (data not shown).

**Figure 4:**
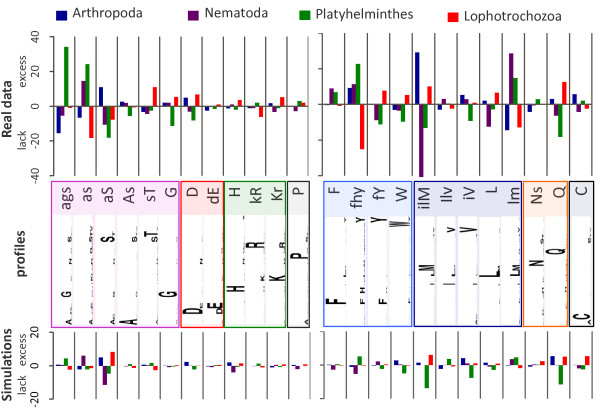
**Excess or lack in number of sites per profile and per clade for the four major clades**. The difference of distribution of the clade-specific profile affiliations is shown for real (top) and the average of ten simulated (bottom) data from the nuc80 dataset; the difference is measured based on the average of sites affiliated to each profile over the four clades. The clades of interest are Arthropoda (blue), Nematoda (purple), Platyhelminthes (green) and Lophotrochozoa (red). Boxes group profiles by similar physico-chemical properties: on the top, the profile name is defined according to the following rules: (i) only the amino acids with a stationary frequency of 0.1 or more are present, (ii) the amino acid is written in uppercase if its stationary frequency is of 0.4 or more; on the bottom, in the profile description, the amino acid is defined by the one-letter code and the height of the letter is proportional to its stationary frequency in the profile.

To further characterize homoplasy in the distribution of profile affiliation, we looked at the distribution of profiles across clades. If the process of change in the substitution process is stationary, one expects a homogeneous distribution. The excess or lack of profile affiliation within a clade is estimated with respect to the average of the distribution among clades under consideration. For the nuclear dataset, the profile distribution is plotted for the four clades of interest; Deuterostomia -used as outgroup- are not shown (Figure 4). Profiles are not equally distributed among clades: some profiles are in excess for one or two clades (e.g. the *ags *profile is more frequent in Platyhelminthes and less frequent in Arthropoda). This unequal distribution of profiles across clades could be studied separately within sub-groups of profiles according to their physico-chemical properties. Three sub-groups (small and uncharged, aromatic, and aliphatic) show a large variation, whereas other sub-groups (in particular, charged amino acids) are more homogeneous. Interestingly, fast evolving Platyhelminthes are the most divergent group, and in the vast majority of cases do not have the same bias as other Lophotrochozoa (Mollusca and Annelida). For instance, for the small and uncharged amino acid sub-group, they are associated preferentially with *ags *or *as *profiles, whereas mollusks and annelids show an affiliation preference for *sT *or *G *profiles. This bias in affiliation frequency across clades generates homoplasy that is difficult to handle, and could explain why Platyhelminthes are so difficult to position (see [27]). Importantly, this heterogeneity is not found in sequences simulated under the CAT model, which is homogenous over time (compare distributions of profile by clade for real and simulated sequences in figure 4), and is significant according to a χ^2 ^test (p-value of 0 and 1 for real and simulated data, respectively). The mt336 dataset yields similar results (Additional file 1: Figure S10).

Since the amino acid composition is influenced by the genomic nucleotide composition [50], heteropecilly could be the result of the heterogeneity of the mutation process over time, i.e. the frequency of some profiles will increase in a clade because their most frequent amino acids are becoming more frequent due to changes in the nucleotide bias. This hypothesis predicts that profiles will be heterogeneously distributed across clades, especially for the fast evolving positions, which are most likely to reflect mutational bias. The profiles are indeed heterogeneously distributed across clades for both mitochondrial and nuclear datasets (Figure 4 and Additional file 1: Figure S10, Additional file 2: Table S8) but, when the fastest evolving positions are considered, this remains highly significant for the mitochondrial alignment only. For this alignment, when one compares an equal number of the most and the least heteropecillous positions, the p-value is slightly lower for the former than for the latter, even if the profiles are significantly heterogeneously distributed across clades. This suggests that changes in the mutational process over time, albeit particularly marked in the mitochondrial genome, are likely a minor cause of heteropecilly.

In summary, the time heterogeneity of the amino acid evolutionary process (heteropecilly) is a general phenomenon in animal evolution, present in both nuclear and mitochondrial coding genomes. Although the rate of change of the substitution process is sufficiently low to allow the recovery of a phylogenetic signal in the recoded sequences, homoplasy is present as suggested by the different rate of evolution across the tree (Figure 3), and by the heterogeneity of profile frequencies across clades (Figure 4 and S10). We do not recommend using the recoding protocol to avoid model violation in cases where heteropecilly is suspected; even if some phylogenetic signal can be captured in the recoded sequences, the signal is probably too weak to obtain an accurate phylogeny.

### Heteropecilly may generate phylogenetic reconstruction artefacts

The observations presented so far demonstrate that the assumption of homopecilly, i.e. no change of the substitution process over time, is violated, potentially leading to tree reconstruction artifacts. As a case study, we chose the relationships between Porifera, Cnidaria and Bilateria (Protostomia+Deuterostomia), which are difficult to resolve with mitochondrial genomes [51]. Since slow-evolving Porifera and Cnidaria are grouped together [52], the monophyly of Eumetazoa (Cnidaria+Bilateria), long proposed by morphologists and recovered with nuclear genes [5], is not observed for the mitochondrial data. We make the assumption that this is due to a long-branch attraction (LBA) artifact between the fast-evolving Bilateria and the distant outgroup (Choanoflagellata). This could be aggravated when using the CAT model by the very long branch observed at the base of Bilateria in recoded sequences (Figure 3), which indicates a large amount of profile affiliation changes, and hence a serious violation of one hypothesis of the CAT model (i.e. the time-homogeneity of the evolutionary process).

**Figure 5:**
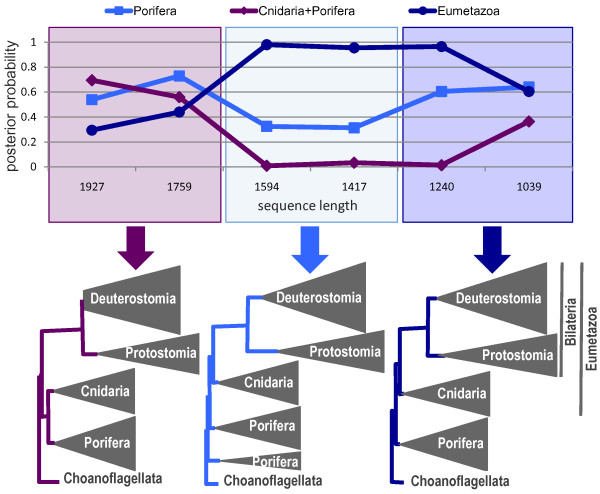
**Topology inferred with a CAT+Γ_4 _model for the mt68 dataset and five sub-alignments after progressive removal of the most heteropecillous sites**. On the top, posterior probabilities for nodes of interest are given for the different sequence lengths.

We analyzed a mitochondrial encoded dataset with 68 species (Additional file 2: Table S3). When using the complete dataset (1927 unambiguously aligned positions), the CAT+Γ_4 _model groups together Cnidaria and Porifera with a posterior probability of 0.70. We then progressively removed heteropecillous positions according to their increasing PIP_n _value; that is, we first removed positions that most likely violate the assumption of homogeneity over time of the CAT model. For the five sub-datasets analyzed with the CAT+Γ_4 _model, three kinds of topologies are observed (Figure 5): (i) with 1759 positions, the same topology as with the complete dataset; (ii) with 1594 and 1417 positions, Eumetazoa (Cnidaria and Bilateria) are recovered, but Porifera are not monophyletic (the homoscleromorphs *Oscarella *and *Plakortis *emerge at the base of Metazoa); (iii) with the smallest subsets, Eumetazoa and Porifera are both monophyletic. With the removal of sites with heterogeneous profile affiliations across clades, support for the monophyly of Eumetazoa (top of Figure 5) increases steadily, up to about one, and decreases to 0.6 in the smallest dataset, probably due to the limited amount of data.

**Figure 6:**
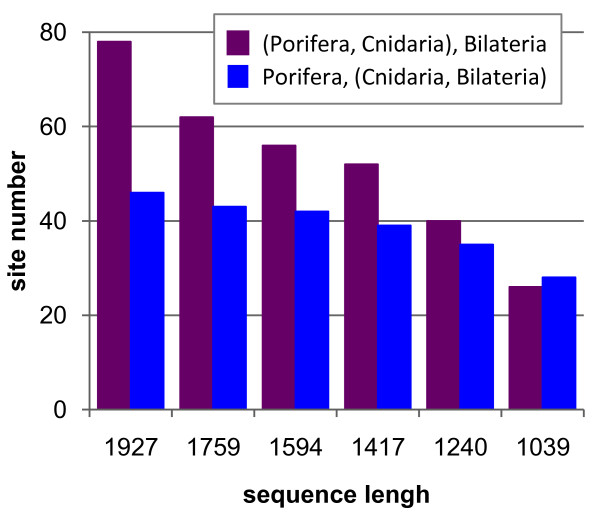
**Distribution of the number of sites grouping preferentially Porifera+Cnidaria or Eumetazoa**. Bars are drawn in purple or in blue, for Porifera+Cnidaria and Eumetazoa, respectively. Sites are counted for the complete alignment and the five subsets obtained by progressive removal of the most heteropecillous sites for the mt68 dataset.

This result suggests that sites showing a substitutional heterogeneity of profiles over time interfere with the phylogenetic signal and may eventually result in an erroneous topology. We computed the number of sites affiliated to the same profile in Cnidaria and Porifera (and to a different one in Bilateria) and in Cnidaria and Bilateria (and to a different one in Porifera). Choanoflagellates are not considered because only two species are available and an accurate profile affiliation to sites is not possible. Figure 6 shows that, upon removal of heteropecillous sites, according to the PIP_n _criterion, the number of sites having the same profile in Porifera and Cnidaria decreases much more rapidly than the number of sites having the same profile in Cnidaria and Bilateria. One can reasonably argue that sites with the same profile in Porifera and Cnidaria generate a spurious signal that is erroneously interpreted by the CAT+Γ_4 _model as synapomorphies for Porifera+Cnidaria, since this model assumes that profiles are identical over the whole tree.

Two controls were performed. First, since a correlation exists between evolutionary rate and heteropecilly (Tables 1 and 2), improvements in phylogenetic inference could be due to the removal of fast evolving sites [12,53]. When fast evolving sites are progressively removed according to the SF method [54], support for the incorrect grouping of Cnidaria and Porifera slightly increases (Additional file 1: Figure S11). This is in sharp contrast with the removal of heteropecillous positions (Figure 5). Second, since the negative effect of heteropecilly may constitute a model violation more important for the CAT+Γ_4 _model than for site-homogeneous models (the site-specific variation of stationary frequencies over time should have less effect -i.e. averaged- when the same stationary frequencies are used for all the sites), the GTR+Γ_4 _or mtREV+Γ_4 _models are also used. Results are completely different: the removal of heteropecillous positions does not affect phylogenetic inferences conducted with GTR+Γ_4 _or mtREV+Γ_4 _models, for which high support for Cnidaria + Porifera is always observed (Additional file 2: Table S9).

This result is not unexpected because, while heteropecilly constitutes a model violation for the CAT model, its effect on the site-homogeneous GTR model is less clear. To understand the different behavior of these two models, one has to distinguish two model violations, heterogeneity of the substitution process across sites and over time. The first violation is known to seriously exacerbate LBA artifacts, because the amount of homoplasy is underestimated [31]. It is not expected to decrease with the removal of heteropecillous positions and probably dominates when using the site-homogeneous GTR and mtREV models. The effect of the second model violation can only be observed with the CAT model, which handles heterogeneity across sites. Removing heteropecillous positions will reduce the time-homogeneous violation and increase the accuracy of the CAT model. This hypothesis is corroborated by an evaluation of model fit by cross-validation (Additional file 2: Table S10). The GTR+Γ_4 _model fits the complete mitochondrial mt68 dataset better than the CAT+Γ_4 _(GTR vs. CAT: 70.7 ± 57.7). This is probably due to the large number of parameters of the latter model and the limited amount of positions, since, with the larger nuclear dataset, the CAT+Γ_4 _model has a better fit than the two models based on exchange matrices (GTR vs. CAT: -1610.3 ± 139.2, WAG vs. CAT: -2615.7 ± 94.4). Importantly, after removal of most heteropecillous sites, the CAT+Γ_4 _model appears to best fit the data (GTR vs. CAT: -45.2 ± 47.0). This indicates that heteropecilly constitutes a serious violation of the CAT model, because the best fit is obtained despite the limited number of sites (1240).

## Conclusion

Numerous heterogeneities of the evolutionary process have been discovered. Most have a clear negative impact on phylogenetic inference when not adequately handled: heterogeneity of rate across lineages [55], of substitution type [56], of rate across sites [57], of composition across taxa [58] and of substitution process across sites [23,24]. Some of them, such as heterotachy, seem to have a more limited effect on phylogenetic accuracy [59]. Further studies are needed to know in which category heteropecilly has to be classified. An important prerequisite is the development of models that handle the fact that substitution properties change over time. This could be achieved via a Markov modulated CAT model, similar to the covarion model [60], or via the use of breakpoints [61]. To choose between these two approaches, it would be important to estimate whether sites generally change their properties in a collective manner or not, since only the second approach can model this aspect. The costs of these improved models (number of parameters, computational time) could be major and would be useful for phylogenetic inference only if heteropecilly turned out to seriously impair accuracy. In any case such improved models would be helpful to advance our knowledge of protein evolution, since in most cases one can select a set of species for whose relationships are confidently known, which drastically simplifies the problem and allows the use of complex but computationally demanding models.

What are the main reasons for these shifts in profile affiliation? The correlation between the PIP_n _criterion and the substitution number might suggest a neutral explanation (e.g. change in mutation pressure) of the variation in substitutional profile over time. Since the PIP_n _criterion is also correlated with a change in hydropathy, this neutral explanation could be insufficient. These changes in the site-specific substitution process could be related to functional shifts, such as adaptation of organisms to new environments (e.g. higher growth temperature) or of the protein to a new cellular environment (e.g. new interactome). To answer these questions, it would be particularly relevant to study paralogous genes in which functional shifts are well characterized.

## Methods

### Sequence Data

Three large datasets have been used: (i) 13 proteins encoded in mitochondria from 336 metazoan species divided into 15 clades (Additional file 2: Table S1), named mt336 dataset, (ii) 111 nucleus encoded proteins from 80 metazoan species divided into 5 clades (Additional file 2: Table S2), named nuc80 dataset, and (iii) 13 mitochondrion encoded proteins from 68 species (66 Metazoa and 2 Choanoflagellata) grouped into 5 clades (Additional file 2: Table S3), named mt68 dataset. All sequences have been downloaded from the GenBank database. For each dataset, proteins have been aligned by ClustalW [62], manually refined using ED [63], and then concatenated into a super-matrix using SCaFoS [64]. Orthology of nuclear proteins was verified using the congruence approach described in [5]. Ambiguously aligned positions have been removed using Gblocks [65], with some manual refinements (necessary because of an inadequate stringency of Gblocks in the presence of missing data). Since we are not interested in constant or quasi-constant positions, which obviously have the same evolutionary properties in all clades, only the parsimony informative positions have been retained, resulting in 1,851, 12,608 and 1,927 positions (from originally 2,547, 22,082 and 2,382 unambiguously aligned positions) for mt336, nuc80 and mt68 datasets, respectively, which allows us to reduce computational costs. The species were selected in order to obtain the most homogeneous taxonomic diversity, that is, monophyletic groups of a similar size (i.e. similar tree length) represented by about twenty species. Two groups have more species (Actinopterygii and Primates) with respect to the tree length criterion.

### Protocol

Topologies were inferred by maximum likelihood separately for each monophyletic group under a WAG [66] or a mtREV [67] model with four gamma discrete categories using Treefinder [68], for the nuc80 and the mitochondrial datasets respectively. As these topologies are biologically reasonable (see Additional file 2: Table S4), all subsequent analyses were performed under these fixed topologies in order to reduce the CPU burden. We verified, in the case of the mt336 dataset, that the same results were obtained under free topology (data not shown).

A scheme of the protocol, described only for two clades for clarity, is shown in the Additional file 1: figure S4. For each clade, the CAT model [24] implemented in the program Phylobayes inferred substitution profiles. However, comparing the profile affiliation across groups is not straightforward since (1) the CAT model infers profiles independently from each clade resulting in different sets of profiles, (2) the number and nature of profiles varies during the MCMC, and (3) different profiles can be affiliated to a given site during the Monte Carlo Markov Chain (MCMC). To achieve our comparison between clades, we need identical profiles in the different runs, and therefore need to define a set of common profiles to which sites can be affiliated whatever the clade. In a first step, the CAT model freely inferred the profiles separately for each clade under a fixed topology; the phylobayes program performed a total of 10,000 cycles, the 1,000 first cycles being discarded as "burn-in". Profiles and their affiliation to positions are repeatedly updated during the MCMC, so different profiles, which are themselves potentially unstable, can be assigned to a same position; we focus on profiles that are the most stable. Stable profiles were identified according to the protocol described in [24] with a threshold value of 0.035 for quadratic distance and a threshold value of 4 for the minimum of profile affiliation to site number. Only profiles that are present >50% of draws from the posterior were retained. Among the stable profiles identified in various clades, some are generally highly similar and need to be further clustered to avoid redundancy. For each pair of profiles, the quadratic distance over the twenty amino acid frequencies was calculated to compute a distance matrix as an input for clustering using UPGMA as implemented in NEIGHBOR [69]. A threshold on the quadratic distance was chosen in order to obtain about 25 clusters (Additional file 1: Figure S1-3), a number of profiles known to provide the greatest step in model fit improvement [70]. Within each cluster, a common profile was defined as the average over the twenty equilibrium frequencies weighted by the affiliation frequency of each initial profile included in the cluster. Twenty-six, twenty-four and twenty-four common profiles were obtained for the large, the small mitochondria encoded proteins and the nuc80 datasets, respectively.

To compare the profile affiliation in different monophyletic groups, phylobayes was re-run with the set of common profiles for each clade separately (CAT model, fixed topology, 1,100 cycles, removing of 100 first cycles). Under these conditions, only the profile affiliations, branch lengths and site-specific rates were free parameters. This allowed to compute p_ik_(c), the posterior probability of affiliation of the profile k to site i for clade c. An affiliation was considered stable if k exists such that p_ik_(c)>0.75.

### Criteria definition

Two criteria have been defined to test the homogeneity of the evolutionary process over time. Homogeneity implies that a given site is affiliated to the same profile in all clades, apart from stochastic fluctuations. For pairwise clade comparison, the Frequency of Different Profiles (FDP) is a global criterion over all the positions for which a profile is stably allocated in the two alignments. The FDP criterion is defined by:

$$\text{FDP}=\frac{{\text{n}}_{\text{dif}}}{{\text{n}}_{\text{dif}}+{\text{n}}_{\text{id}}}$$

where n_dif _is the number of positions with two different profiles in the two clades, and n_id _is the number of positions with two identical profiles. For a threshold of 75% of stability across the MCMC, only 28% and 11% of sites are on average stably affiliated, for the nuc80 dataset and the mt336 dataset respectively. This statistic cannot be extended for comparing all clades simultaneously, since only 1% of the sites are always stably affiliated for the 15 clades of the later dataset. Moreover, the FDP criterion does not give information at the site level.

For the simultaneous comparison of n clades, the Probability of Identical Profiles (PIP_n_) is calculated site by site without any affiliation stability conditions. It is defined for a given site i by:

$${\text{PIP}}_{\text{n}}\text{(i)}=\sum_{\text{k}=1}^{\text{k}}\prod_{\text{c}=1}^{\text{n}}{\text{p}}_{\text{ik}}(\text{c})$$

where K is the number of profiles and n the number of clades. This criterion will take a high value when the site shares the same profiles in different clades. In contrast, a small PIP_n _corresponds to a site that has different evolutionary profiles in the taxonomic groups under consideration. Indeed, even a site with unstable affiliations (i.e. affected to a different set of profiles within a clade) can be compared and shows a PIP_n _value close to zero. For instance, the site can belong to various categories containing hydrophobic profiles in one clade and to various categories containing charged amino acid profiles in other clades. For computational reasons, we have limited the phylobayes runs to 1,100 cycles. This choice increases the number of sites with a PIP_n _value equal to 0: a low frequency of affiliation for a given profile (e.g. 10^-5^) would artificially be estimated at 0 in the posterior distribution. The more cycles performed, the less sites show a zero PIP_n _(Additional file 2: Table S5). However, precision of PIP_n _estimated with 1,000 points is sufficient for the aim of this work (see R^2 ^= 0.999 for comparison between 1000 and 5000 points on Additional file 1: Figure S8). As PIP_n _values are small, in the subsequent analysis, we will use -ln(PIP_n_), except for sites with a PIP_n _value of zero. Because of this latter constraint, results are presented by binning sites into four classes of equal size plus one class for PIP_n _= 0 (Tables 1, 2, 3, 4, 5 and 6, and Additional file 1: Figure S7).

### Evaluation of the protocol

The statistical significance was evaluated by comparing results obtained from real and simulated data. With phylobayes, we performed simulations for each dataset according to the posterior predictive principle, i.e. we took 10 points from the posterior distribution obtained with the real complete dataset (i.e. all species simultaneously) under the CAT+Γ_4 _model (burn-in discarded) and simulated 10 new sequence alignments according to the parameter values of each point, in particular the profiles (for more details see [24,31]). Using the same sets of predefined profiles (obtained from real data), the previously described protocol was used to calculate the FDP and the PIP_n _for each replicate.

Second, we tested that our results were robust vis-à-vis various aspects of our protocol. (i) The use of different stability thresholds yielded virtually identical results in the FDP analysis (Additional file 1: Figure S6B). (ii) To test that low PIP_n _values were due to unstable profile affiliations related to an insufficient number of taxa, we randomly removed half and three quarter of the species in each clade of the large mitochondrial dataset and recomputed PIP_n_. As expected, the less species considered, the less profile affiliations were stable (data not shown), because the phylogenetic signal became insufficient. However, this instability led to a sharp decrease of PIP_n _values equal to 0 (Additional file 1: Figure S5A), indicating that instability was not responsible for low PIP_n _values. (iii) Three additional sets of profiles were used in the case of mt336 alignment: 45 profiles defined using a different threshold on the UPGMA tree, the 25 stable profiles obtained from the analysis of the complete alignment, and the 20 profiles obtained by Le et al. [70]. Similar results were obtained for both PIP_n _(Additional file 1: Figure S5B) and FDP (Additional file 1: Figure S6A) criteria.

Third, we performed a cross-validation test to evaluate the fit of different models on the various datasets: CAT, GTR and WAG/mtREV models were compared using cross-validation as described in Lartillot et al. [31]. An alignment was randomly split in two slices: one tenth for use as a test dataset, and nine-tenths for use as a "training", or "learning" dataset. The parameters were estimated on the learning sets for each model (fixed topology; 21,000 and 11,000 cycles, the first 11,000 and 1,000 cycles discarded, for CAT and others models, respectively) and used to calculate the cross-validation log-likelihood scores of the test sets. Scores were averaged over 10 replicates.

### Influence of profile change on phylogenetic inference

Profiles are representative of the functional constraints acting on a given site in a given clade; if a change of profile occurred in the common ancestor of two clades, the same substitutional profile should be shared by the two sister clades. Hence this can be viewed as a synapomorphy. In other words, the variation of substitution profiles across clades may contain a phylogenetic signal (or noise if the same profile has been independently acquired). A simple recoding approach might capture this putative phylogenetic signal. For reasons of compatibility with available inference tools, each profile is encoded as a one-letter amino acid, therefore only the twenty most frequent profiles have been conserved for this analysis. More precisely, a new sequence is created for each clade according to the following rule: the site is encoded as an amino acid when profile affiliation is stable, and by a question mark otherwise. Under these conditions, the percent of un-encoded sites in the alignments is 59% and 54% for the mt336 and the nuc80 dataset, respectively. It is difficult to know which model of sequence evolution should be used on this artificial alignment. Since we do not know *a priori *which exchangeability rate between profiles should be applied, the resulting file is analyzed with a GTR+Γ_4 _model to infer a phylogenetic tree using phylobayes. To test the effect of the model, we also made inferences with the CAT+Γ_4 _model. To verify the significance of the results, we performed the same analysis with the 10 simulated alignments obtained by using a posterior predictive approach for the mtp336 dataset, as described above.

### Progressive removal of heteropecillous sites

The mt68 dataset was used to evaluate the potential misleading effect of the detected model violations on phylogenetic inference. More precisely, we made the hypothesis that the observed grouping of Cnidaria and Porifera to the exclusion of Bilateria was due to a long branch attraction artifact. We followed the same approach as for heterotachous positions [71,72], by removing the most heterogeneous sites, as estimated by PIP_n_. At each step, we removed ~10% of the positions and stopped when 1,039 positions remained in order to keep a sufficient amount of phylogenetic signal. The five steps corresponded to the exclusion of sites with PIP_n _= 0, and -ln(PIP_n_) higher than 12, 8, 6, and 4.5, respectively. Phylogenetic trees were inferred from these reduced dataset with the CAT+Γ_4 _model using phylobayes. The reduced datasets were also analyzed with GTR+Γ_4 _and mtREV+Γ_4 _models using RAxML [73], the robustness was evaluated with 100 bootstrap replicates.

### Heterotachy analysis

To compare the qualitative heterogeneity studied here (heteropecilly) with the quantitative rate heterogeneity (heterotachy) over time, we looked for heterotachous positions. The number of substitutions per position and per clade was calculated by phylobayes under the CAT+Γ_4 _model. Subsequently, heterotachous positions were identified by the test of Lopez *et al. *[74] (the improved test of Baele et al. [40] is not implemented for amino acid sequences). Eventually, the coefficient of correlation between heterotachy p-values and PIP_n _values over sites was computed.

### Biochemical constraint estimation

We want to know whether the time-variation in profiles corresponds to change in physico-chemical properties of the amino acids involved in the profiles. Profiles were classified into five groups with similar physico-chemical properties (small, aliphatic, aromatic, charged, other) according to the properties of the two amino acids with the highest equilibrium frequencies in the profile. Only sites stably affiliated (threshold = 75%) to two different profiles in clade pairwise comparison were considered. The numbers of sites for which the two profiles were in the same physico-chemical group were counted over all pairwise comparisons.

Finally, we looked for a correlation between heteropecilly and variations in biochemical constraints over time. To do that, we computed a site-specific criterion of hydrophobic variation. For each profile k, a Hydrophobic Score (HS) was computed by summing the hydrophobicity of the twenty amino acids according to Kyte and Doolittle [75] weighted by the equilibrium frequency of the amino acid a_j _in the profile:

$$\text{HS(k)}=\sum_{\text{j}=1}^{20}\pi_{{\text{a}}_{\text{j}}}{\text{(k)*h(a}}_{\text{j}}\text{)}$$

where h(a_j_) is the hydrophobicity of the amino acid a_j _and $\pi_{{\text{a}}_{\text{j}}}$ (k) its equilibrium frequency in the profile k_. _Then, for each clade c and site i, the sitewise Profile Hydrophobic Score (PHS) was calculated by weighting the HS score of each profile k with its affiliation frequency:

$$\text{PHS(c,i)}=\sum_{\text{k}=1}^{\text{k}}{\text{p}}_{\text{ik}}\text{(c)}*\text{HS(k)}$$

To estimate the existence of a hydropathy change over time, the standard deviation of PHS(c,i) across all clades was calculated.

## Authors' contributions

BR made all the experiments, and wrote the first draft of the manuscript. HP conceived and supervised the study. All authors contributed to the analysis of the results and to the writing of the paper. They read and approved the final manuscript.

## Supplementary Material

Additional file 1 **Supplementary figures**. Click here for file

Additional file 2 **Supplementary material**. Click here for file
