# Supplementary material for: Site-specific time heterogeneity of the substitution process and its impact on phylogenetic inference
Source: BMC Evol Biol. 2011 Jan 14;11:17. doi: 10.1186/1471-2148-11-17 (PMC3034684; doi:10.1186/1471-2148-11-17)
Supplement: Additional file 1 — Supplementary figures. [file 1471-2148-11-17-S1.PDF]

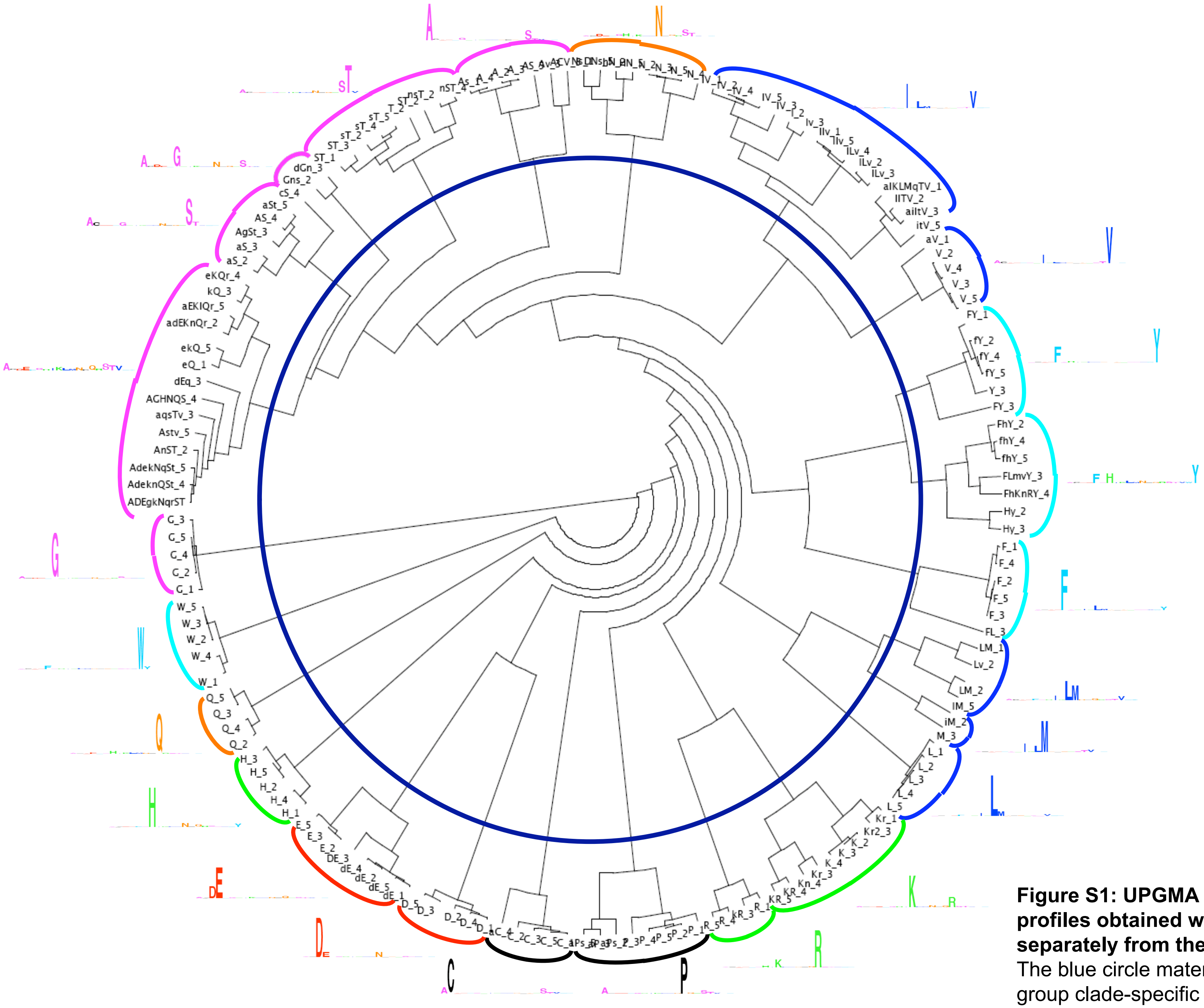

Figure S1: UPGMA tree of substitutional profiles obtained with the CAT model, separately from the five nuc80 clades. The blue circle materializes the threshold to group clade-specific profiles in 24 clustered profiles (i.e. step 2 in figure S4, see Protocol section in Material and methods for details). In description of clustered profile, each amino acid is defined by the one-letter code and the height of the letter is proportional to its stationary frequency. Name of clade-specific profiles is defined according to the following rules: (i) only the amino acids with a stationary frequency of 0.1 or more are present, (ii) the amino acid is written in uppercase if its stationary frequency is of 0.4 or more.

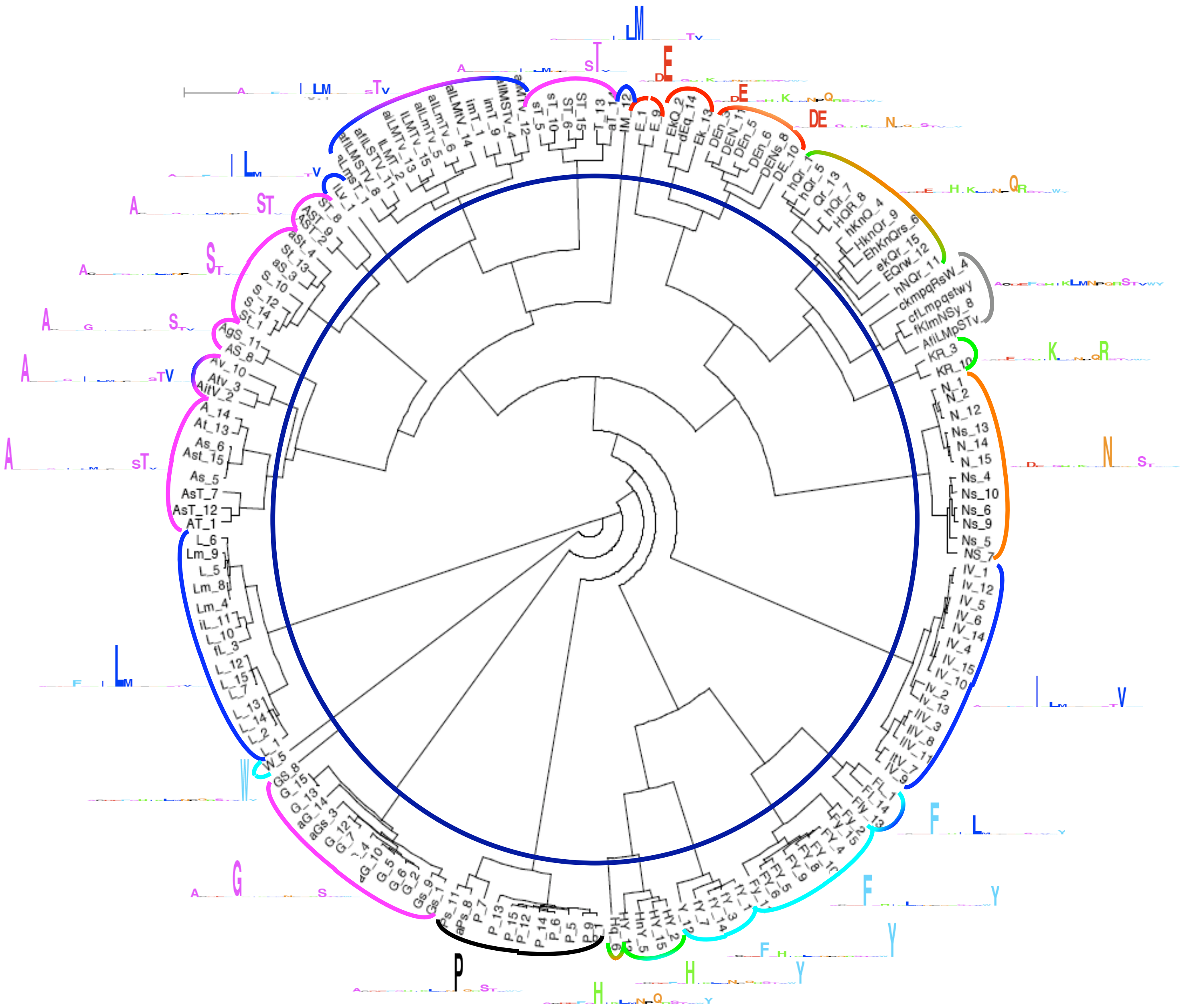

Figure S2: **UPGMA tree of substitutional profiles obtained with the CAT model from the fifteen taxa of the mt336 dataset**. Clustering gives 26 clustered profiles. See figure S1 for legend.

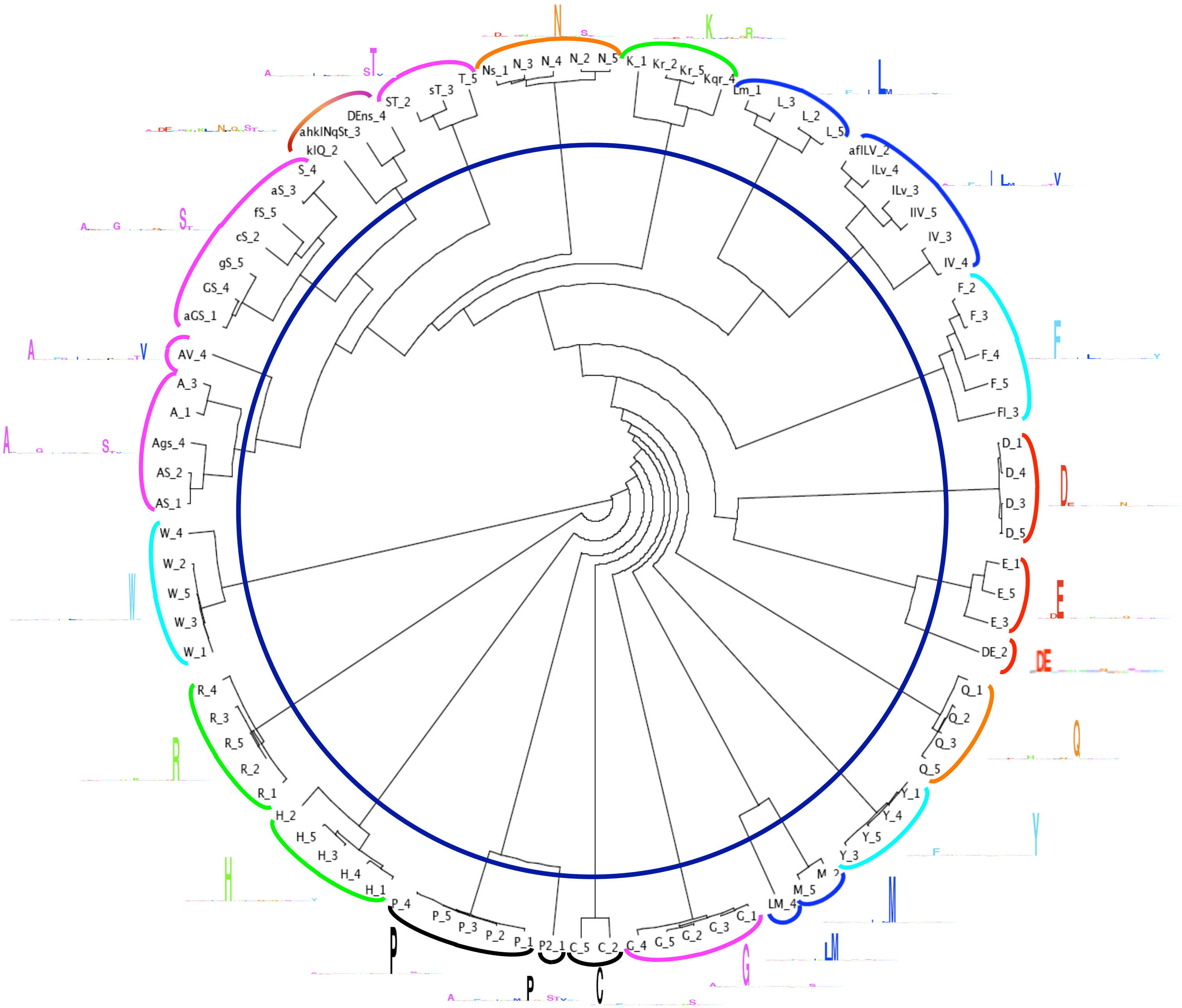

Figure S3: **UPGMA tree of substitutional profiles obtained with the CAT model from the five taxa of the mt68 dataset**. Clustering gives 24 clustered profiles. See figure S1 for legend.

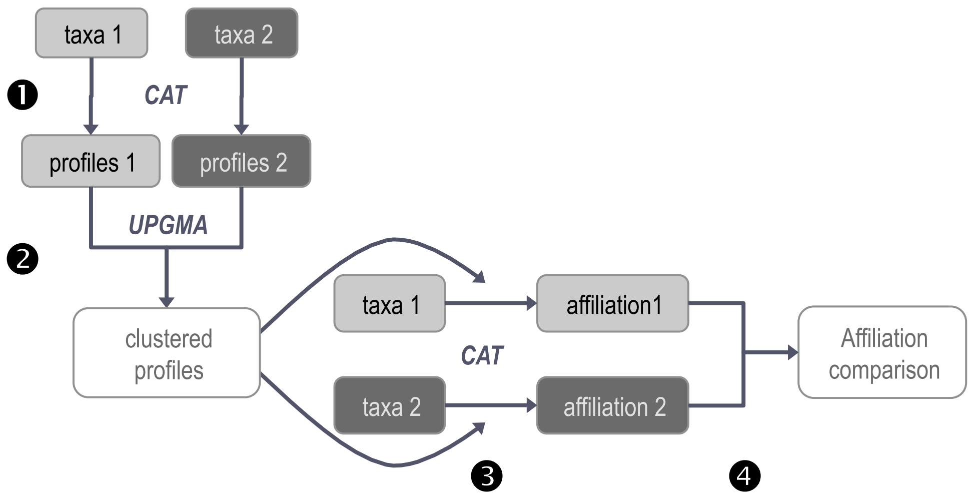

Figure S4: **Plot of the protocol used to compare the profile affiliation in different clades**, the scheme is limited to two taxa for simplification. ① Free inference of subtitutional profiles by the CAT model, separately for each clade. ② Profiles clustering by the UPGMA method. ③ Affiliation of the clustered profiles to sites under the CAT model. ④ Comparison of the affiliation in the different clades.

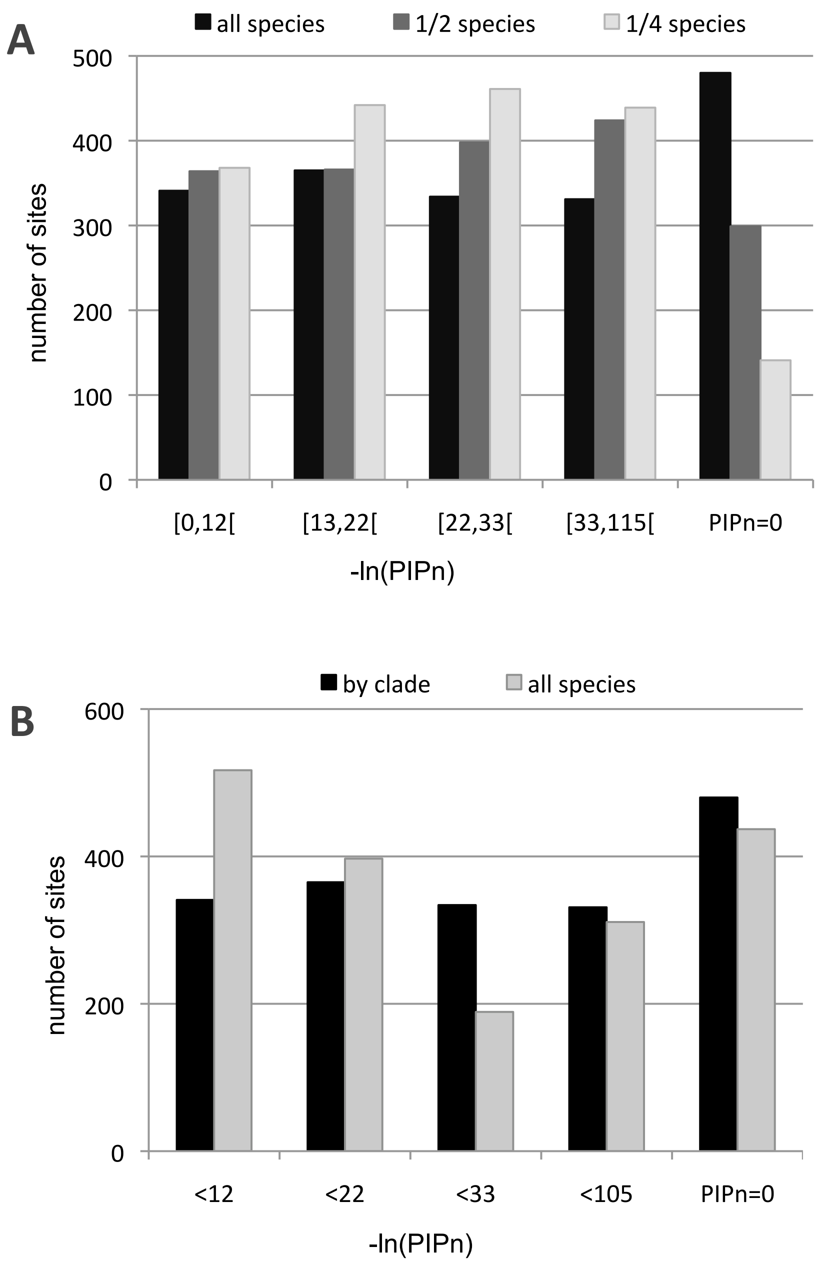

Figure S5: **Distributions of PIPn for different conditions of affiliation on the large mitochondrial dataset.** (A) Affiliation done with three sets of taxa in each clade: all species in black, half the taxon number in dark grey and one quarter of species in light grey. The profile set is the pool of clustered profiles obtained from all species divided in fifteen clades. (B) Comparison of PIPn for two set of profiles: the 26 profiles obtained by clustering, in black, and 25 profiles directly obtained with the 336 species of the complete alignment (in grey).

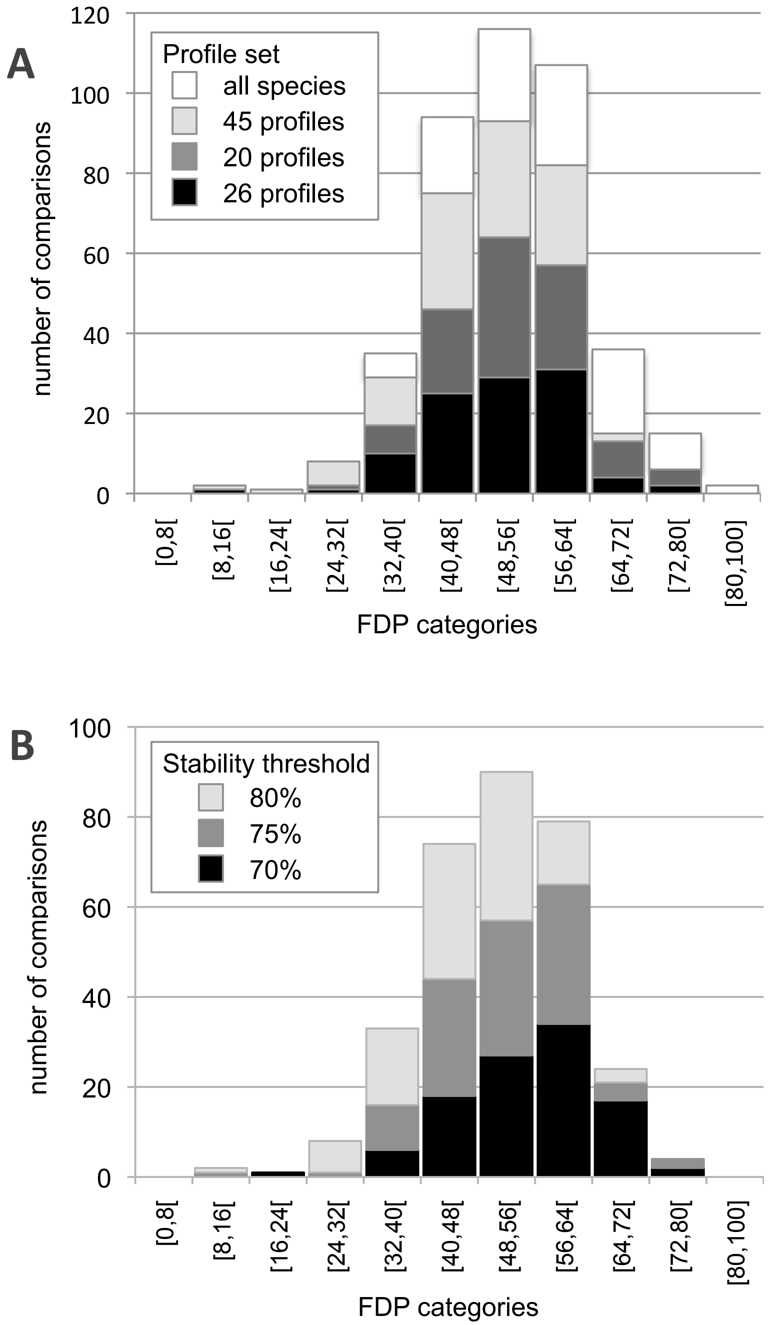

Figure S6: **Stack distributions of FDP values for the large mitochondrial dataset**. (A) Affiliation have been done with four sets of profiles: the 26 profiles used in the analysis, the 20 profiles as defined in Le *et al.*, the 45 profiles clustered with a different threshold on the UPGMA tree and the profiles obtained from all species of the complete alignment: in black, dark grey, medium grey and white, respectively. (B) Using 26 profiles, the stability of the affiliation have been determined according to three affiliation stability threshold values: 70% in black, 75% in dark grey and 80% in medium grey. Plots are drawn for the sites with at least 2 substitutions.

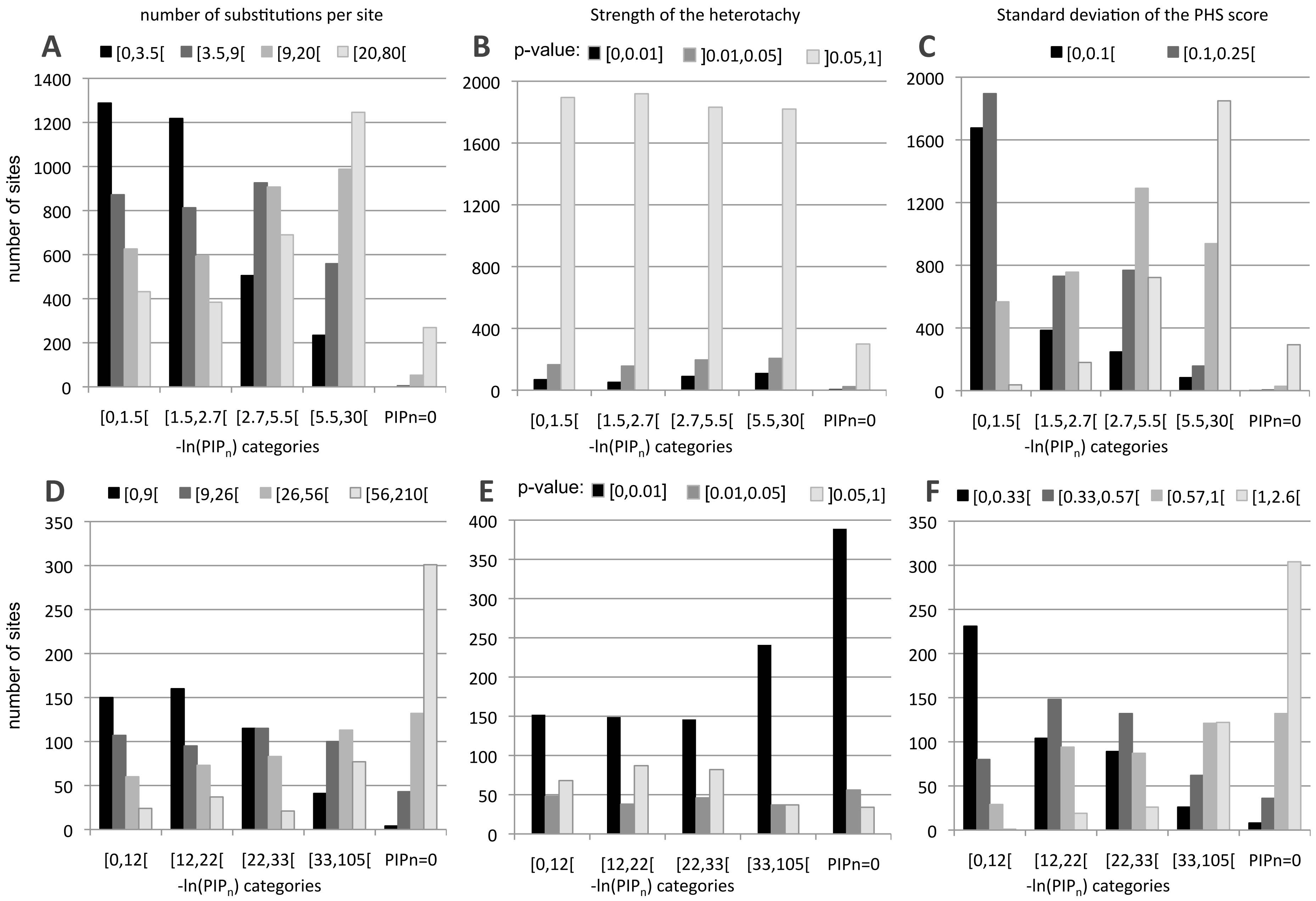

Figure S7: **Distributions of sites based on PIPn values** for the nuclear dataset (top) and the large mitochondrial dataset (bottom). Series are distributed according to the number of substitutions per site (A and D), the strength of the heterotachy (B and E) or the standard deviation of the PHS score (C and F).

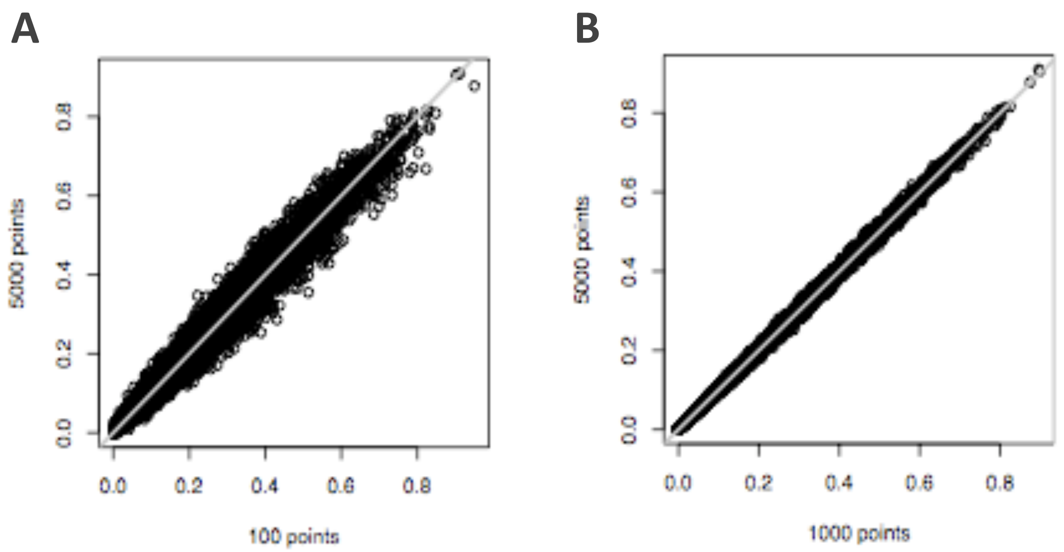

Figure S8: **Dot-plots of PIPn values according to the number of points extracted of the MCMC** after removing of the burn-in (100 first points): (A) 100 points against 5000 points, (B) 1000 points against 5000 points. The regression curve is plotted in grey.

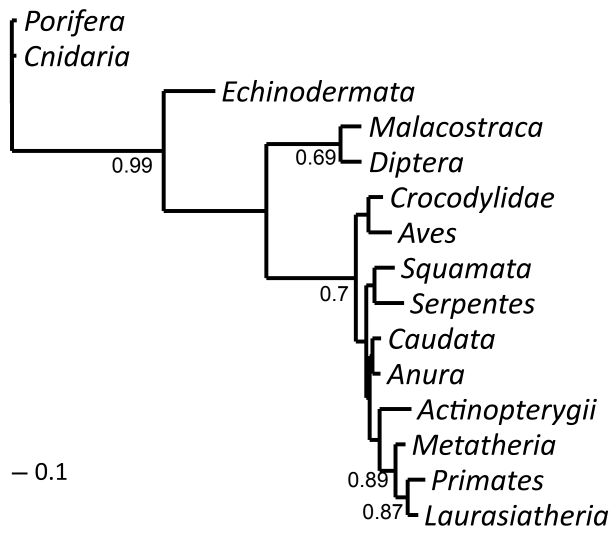

Figure S9: **Topology inferred with a CAT+Γ_4 model from the mt336 dataset recoded bystable profiles**. Only posterior probabilities greater or equal to 0.5 presented.

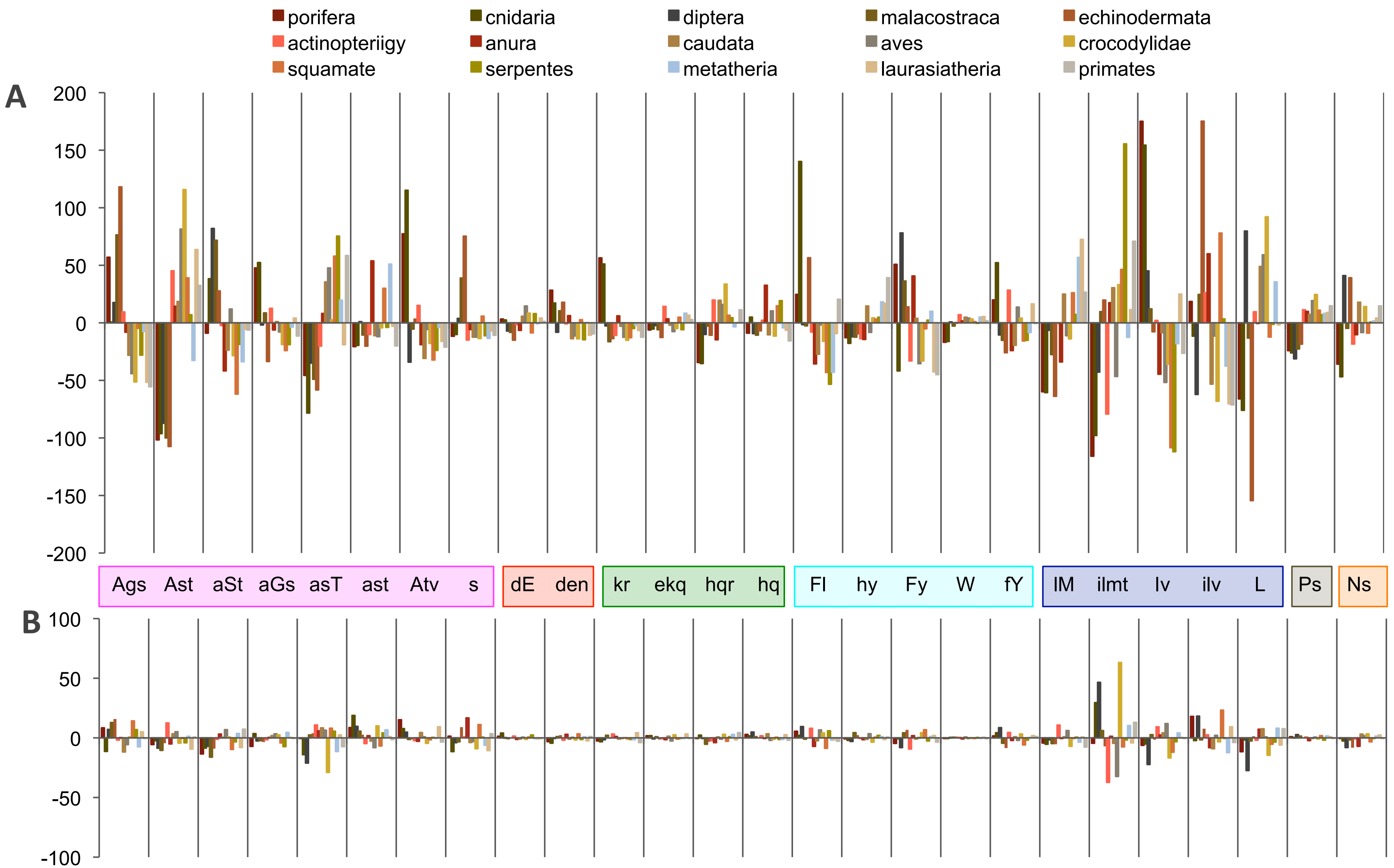

Figure S10: **Excess or lack in number of sites per profiles and per taxa the mt336 dataset**. The distribution is sorted by profile for real (A) and average of ten simulated data (B); the difference is measured based on the average of sites affiliated to each profile over the fifteen taxa. Profile names defined as in figure 4. Colored boxes group profiles with similar physico-chemical properties (small, negatively charged, positively charged, aromatic, aliphatic, other properties).

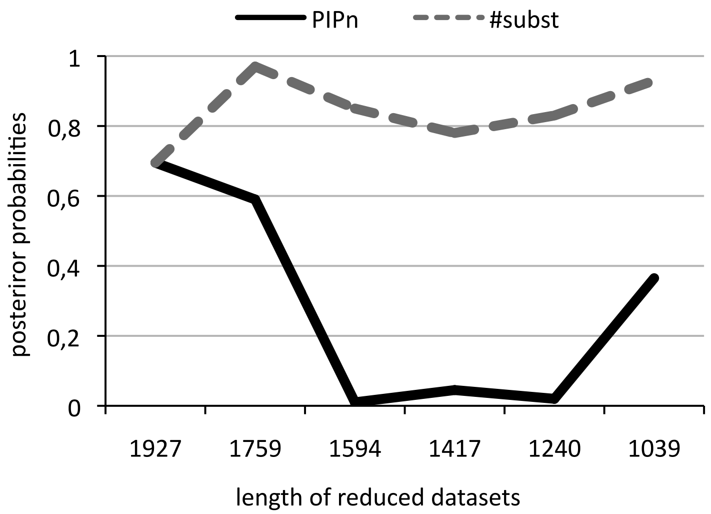

Figure S11: **Posterior probabilities observed for the node grouping Cnidaria and Porifera after removal of most heteropecillous sites** (in black) or fastest sites (in grey dots). Phylogenetic inferences where done with the CAT+Γ_4 model using Phylobayes.
