# Supplementary material for: Site-specific time heterogeneity of the substitution process and its impact on phylogenetic inference
Source: BMC Evol Biol. 2011 Jan 14;11:17. doi: 10.1186/1471-2148-11-17 (PMC3034684; doi:10.1186/1471-2148-11-17)
Supplement: Additional file 2 — Supplementary material. [file 1471-2148-11-17-S2.PDF]

Site-specific time heterogeneity of the substitution process and its impact on the phylogenetic inference

Béatrice ROURE and Hervé PHILIPPE

Département de Biochimie, Centre Robert-Cedergren, Université de Montréal,

Supplementary material

Table S1: Species list by taxon for the mt336 dataset. In parenthesis, the species number. The access number is indicated for each mitochondrial genome.

**actinopterygii (42)**	
Abudefduf vaigiensis	NC_009064
Astronotus ocellatus	NC_009058
Neolamprologus brichardi	NC_009062
Cymatogaster aggregata	NC_009059
Labracinus cyclophthalmus	NC_009054
Acanthurus leucosternon	NC_009830
Monodactylus argenteus	NC_009858
Antigonia capros	NC_003191
Plectropomus leopardus	NC_008449
Lethrinus obsoletus	NC_009855
Pagrus major	NC_003196
Oplegnathus fasciatus	DQ872160
Anarhichas denticulatus	EF427918
Percina macrolepida	NC_008111
Halichoeres melanurus	NC_009066
Pseudolabrus sieboldi	NC_009067
Scomber scombrus	NC_006398
Thunnus orientalis	NC_008455
Carangoides armatus	NC_004405
Lates calcarifer	NC_007439
Chaetodontoplus septentrionalis	NC_009873
Acheilognathus typus	NC_008668
Tinca tinca	NC_008648
Campostoma anomalum	NC_008102
Pseudaspius leptocephalus	NC_008681
Zacco sieboldii	NC_008653
Gobio gobio	NC_008662
Hemibarbus barbus	NC_008644
Danio rerio	NC_002333
Labeo senegalensis	NC_008657
Puntius ticto	NC_008658
Crossostoma lacustre	NC_001727
Minytrema melanops	NC_008113
Leptobotia mantschurica	NC_008677
Schistura balteata	NC_008679
Pangio anguillaris	NC_008675
Chanos chanos	NC_004693
Grasseichthys gabonensis	NC_007890
Chalceus macrolepidotus	NC_004700
Pangasianodon gigas	NC_006381
Corydoras rabauti	NC_004698
Eigenmannia sp.	NC_004701

**anura (21)**	
Alytes obstetricans pertinax	NC_006688
Bombina orientalis	AY957562
Bombina variegata	NC_009258
Discoglossus galganoi	NC_006690
Xenopus laevis	NC_001573
Xenopus tropicalis	NC_006839
Pelobates cultripes	NC_008144
Amolops tormotus	NC_009423
Rana nigromaculata	NC_002805
Limnonectes fujianensis	NC_007440
Buergeria buergeri	NC_008975
Polypedates megacephalus	NC_006408
Rhacophorus schlegelii	NC_007178
Mantella madagascariensis	NC_007888
Kaloula pulchra	NC_006405
Microhyla heymonsi	NC_006406
Microhyla ornata	NC_009422
Bufo gargarizans	NC_008410
Bufo melanostictus	NC_005794
Hyla chinensis	NC_006403
Hyla japonica	NC_010232

**caudata (21)**	
Ambystoma laterale	NC_006330
Lyciasalamandra atifi	NC_002756
Aneides hardii	NC_006338
Desmognathus fuscus	NC_006339
Phaeognathus hubrichti	NC_006344
Ensatina eschscholtzii	NC_006328
Plethodon elongatus	NC_006335
Batrachoseps wrightorum	NC_006333
Nototriton abscondens	AY728229
Eurycea bislineata	NC_006329
Rhyacotriton variegatus	NC_006331
Andrias davidianus	NC_004926
Onychodactylus fischeri	NC_008089
Batrachuperus yenyuanensis	DQ333818
Pseudohynobius tsinpaensis	NC_008081
Salamandrella keyserlingii	NC_008082
Hynobius amjiensis	NC_008076
Hynobius leechii	NC_008079
Hynobius arisanensis	NC_009335
Pachyhynobius shangchengensis	NC_008080
Ranodon sibiricus	NC_004021

serpentes (13)	
Acrochordus granulatus	NC_007400
Agkistrodon piscivorus	NC_009768
Ovophis okinavensis	NC_007397
Deinagkistrodon acutus	NC_010223
Dinodon semicarinatus	NC_001945
Pantherophis slowinskii	NC_009769
Enhydris plumbea	NC_010200
Boa constrictor	NC_007398
Cylindrophis ruffus	NC_007401
Python regius	NC_007399
Xenopeltis unicolor	NC_007402
Leptotyphlops dulcis	NC_005961
Ramphotyphlops braminus	NC_010196

laurasiatheria (37)	
Ailuropoda melanoleuca	EF196663
Tremarctos ornatus	NC_009969
Ursus arctos	NC_003427
Ursus thibetanus	NC_009971
Ailurus fulgens	AM711897
Spilogale putorius	AM711898
Enhydra lutris	NC_009692
Martes melampus	NC_009678
Meles meles	AM711900
Canis familiaris	AY729880
Vulpes vulpes	NC_008434
Procyon lotor	NC_009126
Arctocephalus forsteri	NC_004023
Zalophus californianus	NC_008416
Callorhinus ursinus	NC_008415
Odobenus rosmarus rosmarus	NC_004029
Erignathus barbatus	NC_008426
Phoca caspica	NC_008431
Monachus schauinslandi	NC_008421
Mirounga leonina	NC_008422
Felis catus	NC_001700
Neofelis nebulosa	NC_008450
Herpestes javanicus	NC_006835
Balaena mysticetus	AP006472
Balaenoptera bonaerensis	NC_006926
Caperea marginata	NC_005269
Hyperoodon ampullatus	NC_005273
Lagenorhynchus albirostris	NC_005278
Lipotes vexillifer	NC_007629
Kogia breviceps	NC_005272
Bos taurus	AF492351
Cervus nippon centralis	NC_006993
Muntiacus reevesi	NC_004069
Ovis aries	NC_001941
Camelus dromedarius	NC_009849
Lama pacos	AJ566364
Sus scrofa	NC_000845

squamata (20)	
Abronia graminea	NC_005958
Heloderma suspectum	NC_008776
Shinisaurus crocodilurus	NC_005959
Iguana iguana	NC_002793
Sceloporus occidentalis	NC_005960
Lacerta viridis viridis	NC_008328
Takydromus tachydromoides	NC_008773
Cordylus warreni	NC_005962
Lepidophyma flavimaculatum	NC_008775
Plestiodon egregius	NC_000888
Coleonyx variegatus	NC_008774
Gekko vittatus	NC_008772
Heteronotia binoei	EF626808
Teratoscincus keyserlingii	NC_007008
Amphisbaena schmidti	NC_006284
Geocalamus acutus	NC_006285
Diplometopon zarudnyi	NC_006283
Bipes biporus	AY605481
Bipes canaliculatus	NC_006288
Bipes tridactylus	NC_006286

metatheria (24)	
Caenolestes fuliginosus	NC_005828
Rhyncholestes raphanurus	NC_005829
Dactylopsila trivirgata	NC_008134
Pseudocheirus peregrinus	NC_006519
Petaurus breviceps	NC_008135
Distoechurus pennatus	NC_008145
Tarsipes rostratus	NC_006518
Lagostrophus fasciatus	NC_008447
Macropus robustus	NC_001794
Potorous tridactylus	NC_006524
Phalanger interpositus	NC_008137
Trichosurus vulpecula	NC_003039
Vombatus ursinus	NC_003322
Dromiciops gliroides	AJ508402
Echymipera rufescens australis	NC_007632
Perameles gunnii	NC_006521
Macrotis lagotis	NC_006520
Didelphis virginiana	NC_001610
Thylamys elegans	NC_005825
Monodelphis domestica	NC_006299
Dasyurus hallucatus	NC_007630
Phascogale tapoatafa	NC_006523
Sminthopsis douglasi	NC_006517
Notoryctes typhlops	NC_006522

primates (25)	
Chlorocebus aethiops	NC_007009
Chlorocebus sabaeus	EF597503
Macaca mulatta	NC_005943
Macaca sylvanus	NC_002764
Papio hamadryas	NC_001992
Colobus guereza	NC_006901
Procolobus badius	NC_008219
Nasalis larvatus	NC_008216
Pygathrix roxellana	NC_008218
Pygathrix nemaeus	NC_008220
Presbytis melalophos	NC_008217
Trachypithecus obscurus	NC_006900
Semnopithecus entellus	NC_008215
Gorilla gorilla	NC_001645
Homo sapiens	AY195791
Pan troglodytes	NC_001643
Pongo abelii	NC_002083
Pongo pygmaeus	NC_001646
Hylobates lar	NC_002082
Cebus albifrons	NC_002763
Daubentonia madagascariensis	NC_010299
Eulemur mongoz	NC_010300
Lemur catta	NC_004025
Nycticebus coucang	NC_002765
Tarsius bancanus	NC_002811

echinodermata (18)	
Acanthaster brevispinus	NC_007789
Acanthaster planci	NC_007788
Patiria pectinifera	NC_001627
Astropecten polyacanthus	NC_006666
Luidia quinalia	NC_006664
Asterias amurensis	NC_006665
Pisaster ochraceus	NC_004610
Cucumaria miniata	NC_005929
Arbacia lixula	NC_001770
Paracentrotus lividus	NC_001572
Strongylocentrotus droebachiensis	NC_009940
Strongylocentrotus purpuratus	NC_001453
Strongylocentrotus pallidus	NC_009941
Florometra serratissima	NC_001878
Phanogenia gracilis	NC_007690
Gymnocrinus richeri	NC_007689
Ophiopholis aculeata	NC_005334
Ophiura lutkeni	NC_005930

malacostraca (19)	
Callinectes sapidus	NC_006281
Portunus trituberculatus	NC_005037
Pseudocarcinus gigas	NC_006891
Eriocheir sinensis	NC_006992
Geothelphusa dehaani	NC_007379
Pagurus longicarpus	NC_003058
Cherax destructor	NC_011243
Panulirus japonicus	NC_004251
Fenneropenaeus chinensis	NC_009679
Penaeus monodon	NC_002184
Litopenaeus vannamei	DQ534543
Marsupenaeus japonicus	NC_007010
Halocaridina rubra	NC_008413
Macrobrachium rosenbergii	NC_006880
Gonodactylus chiragra	NC_007442
Harpiosquilla harpax	NC_006916
Squilla empusa	NC_007444
Squilla mantis	NC_006081
Lysiosquillina maculata	NC_007443

diptera (18)	
Aedes aegypti	NC_010241
Aedes albopictus	NC_006817
Anopheles gambiae	NC_002084
Anopheles quadrimaculatus A	NC_000875
Bactrocera carambolae	NC_009772
Bactrocera oleae	NC_005333
Ceratitis capitata	NC_000857
Chrysomya putoria	NC_002697
Cochliomyia hominivorax	NC_002660
Haematobia irritans irritans	NC_007102
Dermatobia hominis	NC_006378
Drosophila melanogaster	NC_001709
Drosophila sechellia	AF200832
Drosophila yakuba	NC_001322
Simosyrphus grandicornis	NC_008754
Trichophthalma punctata	NC_008755
Cydistomyia duplonotata	NC_008756
Culicoides arakawae	NC_009809

crocodylidae (11)	
Alligator mississippiensis	NC_001922
Alligator sinensis	NC_004448
Caiman crocodilus	NC_002744
Paleosuchus palpebrosus	AM493870
Paleosuchus trigonatus	NC_009732
Crocodylus niloticus	NC_008142
Crocodylus porosus	NC_008143
Crocodylus siamensis	NC_008795
Crocodylus siamensis	EF581859
Osteolaemus tetraspis	NC_009728
Gavialis gangeticus	NC_008241

porifera (17)	
Amphimedon compressa	NC_010201
Amphimedon queenslandica	NC_008944
Callyspongia plicifera	NC_010206
Xestospongia muta	NC_010211
Axinella corrugata	NC_006894
Iotrochota birotulata	NC_010207
Negombata magnifica	NC_010171
Rhabdocalyptus dawsoni	NC_009627
Tethya actinia	NC_006991
Topsentia ophiraphidites	NC_010204
Geodia neptuni	NC_006990
Ephydatia muelleri	NC_010202
Aplysina fulva	NC_010203
Chondrilla aff. Nucula CHOND	NC_010208
Halisarca dujardini	NC_010212
Oscarella carmela	NC_009090
Plakortis angulospiculatus	NC_010217

cnidaria (15)	
Acropora tenuis	NC_003522
Montipora cactus	NC_006902
Agaricia humilis	NC_008160
Siderastrea radians	NC_008167
Porites porites	NC_008166
Rhodactis sp.CASIZ.171755	NC_008158
Astrangia sp.JVK.2006	NC_008161
Colpophyllia natans	NC_008162
Montastraea franksi	NC_007225
Pocillopora eydouxi	NC_009798
Seriatopora caliendrum	NC_010245
Chrysopathes formosa	NC_008411
Metridium senile	NC_000933
Nematostella sp.JVK.2006	NC_008164
Savalia savaglia	NC_008827

Table S2: Species list by taxon for the nuc80 dataset. In parenthesis, the species number.

annelids and molluscs (16)
Chaetopterus sp
Platynereis dumerilii
Tubifex tubifex
Lumbricus rubellus
Hirudo medicinalis
Helobdella robusta
Capitella sp i ecs-2004
Euprymna scolopes
Venerupis decussatus
Crassostrea gigas
Mytilus galloprovincialis
Argopecten irradians
Lottia gigantea
Lymnaea stagnalis
Biomphalaria glabrata
Aplysia californica

nematods (16)
Trichinella spiralis
Onchocerca volvulus
Brugia malayi
Ascaris suum
Strongyloides ratti
Meloidogyne incognita
Radopholus similis
Heterodera glycines
Globodera rostochiensis
Bursaphelenchus xylophilus
Pristionchus pacificus
Caenorhabditis briggsae
Caenorhabditis elegans
Heterorhabditis bacteriophora
Haemonchus contortus
Ancylostoma caninum

arthropoda (21)
Lepeophtheirus salmonis
Litopenaeus vannamei
Petrolisthes cinctipes
Carcinus maenas
Daphnia pulex
Artemia franciscana
Onychiurus arcticus
Pediculus humanus
Rhodnius prolixus
Nilaparvata lugens
Locusta migratoria
Gryllus bimaculatus
Tribolium castaneum
Diabrotica virgifera
Spodoptera frugiperda
Bombyx mori
Nasonia vitripennis
Apis mellifera
Ixodes scapularis
Boophilus microplus
Acanthoscurria gomesiana

platyhelmintes (9)
Macrostomum lignano
Schistosoma japonicum
Schistosoma mansoni
Opisthorchis viverrini
Fasciola hepatica
Taenia solium
Echinococcus granulosus
Schmidtea mediterranea
Dugesia ryukyuensis

deuterostomia (18)
Xenoturbella bocki
Saccoglossus kowalevskii
Ptychodera flava
Strongylocentrotus purpuratus
Asterina pectinifera
Branchiostoma floridae
Molgula tectiformis
Halocynthia roretzi
Ciona savignyi
Ciona intestinalis
Petromyzon marinus
Eptatretus burgeri
Squalus acanthias
Tetraodon nigroviridis
Danio rerio
Homo sapiens
Xenopus tropicalis
Ambystoma mexicanum

Table S3: Species list by taxa for the mt68 dataset. In parenthesis, the species number.

**Porifera (23)**	
Agelas schmidti	EU237475
Amphimedon compressa	NC 010201
Amphimedon queenslandica	NC 008944
Aplysina fulva	NC 010203
Axinella corrugata	NC 006894
Callyspongia plicifera	NC 010206
Chondrilla aff. Nucula CHOND	NC 010208
Cinachyrella kuekenthali	EU237479
Ectyoplasia ferox	EU237480
Ephydatia muelleri	NC 010202
Geodia neptuni	NC 006990
Halisarca dujardini	NC 010212
Igernella notabilis	NC 010216
Iotrochota birotulata	NC 010207
Negombata magnifica	NC 010171
Oscarella carmela	NC 009090
Plakortis angulospiculatus	NC 010217
Ptilocaulis walpersi	EU237488
Rhabdocalyptus dawsoni	NC 009627
Suberites domuncula	NC 010496
Tethya actinia	NC 006991
Topsentia ophiraphidites	NC 010204
Xestospongia muta	NC 010211

**Cnideria (15)**	
Astrangia sp.JVK.2006	NC 008161
Chrysopathes formosa	NC 008411
Colpophyllia natans	NC 008162
Discosoma sp.CASIZ.16891	NC 008072
Madracis mirabilis	NC 011160
Metridium senile	NC 000933
Montastraea franksi	NC 007225
Montipora cactus	NC 006902
Nematostella sp.JVK.2006	NC 008164
Pavona clavus	NC 008165
Pocillopora eydouxi	NC 009798
Porites porites	NC 008166
Ricordea florida	NC 008159
Savalia savaglia	NC 008827
Siderastrea radians	NC 008167

**Protostomia (15)**	
Adoxophyes honmai	NC 008141
Epiperipatus biolleyi	NC 009082
Limulus polyphemus	NC 003057
Loxocorone allax	NC 010431
Lumbricus terrestris	NC 001673
Metaperipatus inae	NC 010961
Penaeus monodon	NC 002184
Pista cristata	NC 011011
Platynereis dumerilii	NC 000931
Priapulus caudatus	NC 008557
Scutigera coleoptrata	NC 005870
Sipunculus nudus	NC 011826
Trachypachus holmbergi	NC 011329
Triops longicaudatus	NC 006079
Urechis caupo	NC 006379

**Deutetostomia (13)**	
Asymmetron lucayanum	NC 006464
Balanoglossus carnosus	NC 001887
Branchiostoma belcheri	NC 004537
Cucumaria miniata	NC 005929
Gymnocrinus richeri	NC 007689
Latimeria chalumnae	AB257297
Lepidosiren paradoxa	NC 003342
Ophiura lutkeni	NC 005930
Petromyzon marinus	NC 001626
Pisaster ochraceus	NC 004610
Saccoglossus kowalevskii	NC 007438
Squalus acanthias	NC 002012
Strongylocentrotus droebachiensis	NC 009940

**Choanoflagellata (2)**	
Capsaspora owczarzaki	MBE(2008) **25**:664-72
Monosiga brevicolis	NC_004309

Table S4: Unrooted species trees for the three datasets.

mt336 dataset

(Abronia_gr:0.264,Heloderma_:0.197,(Shinisauru:0.160,((Lepidophym:0.255,Cordylus_w:0.2375):0.0355,((Takydromus:0.111,La
certa_vi:0.101):0.145,(((Bipes_trid:0.132,(Bipes_cana:0.0990,Bipes_bipo:0.085):0.040):0.235,(Diplometop:0.170,(Geocalamus:0.
149,Amphisbaen:0.152):0.064):0.035):0.187,(Ramphotyph:0.494,(Leptotyphl:0.496,((((Xenopeltis:0.068,Python_reg:0.084):0.022
,Cylindroph:0.097):0.021,Boa_constr:0.124):0.022,(((Enhydris_p:0.16,(Pantheroph:0.049,Dinodon_se:0.061):0.074):0.031,((Ovop
his_ok:0.05,Deinagkist:0.063):0.016,Agkistrodo:0.041):0.085):0.052,Acrochordu:0.221):0.041):0.416):0.082):0.558):0.06):0.047,((
Plestiodon:0.126,(Sceloporus:0.104,Iguana_igu:0.084):0.054):0.020,(((Teratoscin:0.192,(Heteronoti:0.212,Gekko_vitt:0.19):0.078
):0.056,Coleonyx_v:0.150):0.0858,((((Gavialis_g:0.059,(Osteolaemu:0.083,((Crocodylus:0.021,Crocodyl02:0.017):0.0059,(Croco
dyl01:0.0072,Crocodyl00:0.005):0.024):0.037):0.067):0.144,(((Paleosuchu:0.039,Paleosuc00:0.041):0.058,Caiman_cro:0.146):0.
065,(Alligator_:0.071,Alligato00:0.079):0.033):0.075):0.485,(Meleagris_:0.076,(Alectura_l:0.046,((Cygnus_col:0.071,Anseranas_:
0.032):0.017,((Struthio_c:0.039,(((Tinamus_ma:0.096,Eudromia_e:0.067):0.038,Dinornis_g:0.036):0.018,((Pterocnemi:0.042,Cas
uarius_:0.023):0.009,Apteryx_ha:0.04):0.0052):0.007):0.038,(((Pterogloss:0.081,Dryocopus_:0.062):0.026,Archilochu:0.069):0.02
,(((Strigops_h:0.041,Melopsitta:0.063):0.047,(Gavia_paci:0.036,((Phaethon_r:0.060,Ninox_nova:0.114):0.018,((Micrastur_:0.048,
Falco_spar:0.082):0.026,(Eudyptes_c:0.041,Buteo_bute:0.068):0.0075):0.006):0.0053):0.005):0.007,(Ciconia_bo:0.035,(Podicep
s_c:0.049,Arenaria_i:0.035):0.008):0.005,(Fregata_sp:0.043,(Platalea_m:0.027,((Smithornis:0.133,Cnemotricc:0.062):0.019,(Me
nura_nov:0.06,(Corvus_fru:0.057,((Taeniopygi:0.04,Sylvia_cra:0.068):0.017,Acrocephal:0.047):0.021):0.027):0.062):0.029):0.006
):0.0056):0.008):0.01):0.027):0.021):0.037):0.210):0.097,(((((Monodelphi:0.053,(Thylamys_e:0.073,Didelphis_:0.087):0.018):0.03
3,(((Notoryctes:0.129,(Macrotis_l:0.05,(Perameles_:0.034,Echymipera:0.032):0.016):0.027):0.011,(Dromiciops:0.069,(Sminthopsi
:0.046,(Phascogale:0.046,Dasyurus_h:0.068):0.018):0.055):0.012):0.0069,((Vombatus_u:0.066,((Trichosuru:0.031,Phalanger_:0.
08):0.023,(Potorous_t:0.033,(Macropus_r:0.027,Lagostroph:0.022):0.012):0.02):0.01):0.01,(Distoechur:0.062,(Tarsipes_r:0.077,(
Pseudochei:0.044,Petaurus_b:0.064,Dactylopsi:0.071):0.0084):0.007):0.009):0.009):0.011):0.013,(Rhyncholes:0.041,Caenoleste
:0.039):0.042):0.097,((Tarsius_ba:0.096,((Nycticebus:0.144,((Lemur_catt:0.028,Eulemur_mo:0.033):0.059,Daubentoni:0.132):0.0
14):0.028,(((Hylobates_:0.061,((Pongo_pygm:0.0296,Pongo_abel:0.0227):0.066,((Pan_troglo:0.022,Homo_sapie:0.029):0.017,G
orilla_go:0.037):0.025):0.0234):0.04,((((Semnopithe:0.058,(Trachypith:0.042,Presbytis_:0.053):0.008):0.012,(Pygathri00:0.043,(P
ygathrix_:0.036,Nasalis_la:0.038):0.01):0.009):0.015,(Procolobus:0.046,Colobus_gu:0.0603):0.028):0.032,((Papio_hama:0.0747,
(Macaca_syl:0.054,Macaca_mul:0.046):0.038):0.028,(Chlorocebu:0.018,Chloroce00:0.023):0.055):0.047):0.063):0.079,Cebus_al
bi:0.28):0.112):0.017):0.035,(((Sus_scrofa:0.071,(Lama_pacos:0.03,Camelus_dr:0.0369):0.065):0.013,(((Ovis_aries:0.037,(Munti
acus_:0.012,Cervus_nip:0.021):0.017):0.009,Bos_taurus:0.031):0.028,((Kogia_brev:0.065,((Lipotes_ve:0.097,La9enorhyn:0.058)
:0.028,Hyperoodon:0.044):0.011):0.008,(Balaenopte:0.026,(Caperea_ma:0.032,Balaena_my:0.016):0.005):0.021):0.084):0.009):
0.021,((Herpestes_:0.059,(Neofelis_n:0.031,Felis_catu:0.019):0.024):0.020,(((Mirounga_l:0.026,(Monachus_s:0.036,(Phoca_cas
p:0.018,Erignathus:0.017):0.01):0.005):0.012,(Odobenus_r:0.078,(Callorhinu:0.028,(Zalophus_c:0.012,Arctocepha:0.02):0.012):0
.028):0.024):0.019,((Vulpes_vul:0.02,Canis_fami:0.022):0.034,(((Spilogale_:0.063,Procyon_lo:0.079):0.01,((Meles_mele:0.04,(M
artes_mel:0.028,Enhydra_lu:0.041):0.008):0.021,Ailurus_fu:0.056):0.009):0.008,(((Ursus_thib:0.018,Ursus_arct:0.02):0.017,Trem
arctos:0.049):0.013,Ailuropoda:0.045):0.019):0.005):0.009):0.017):0.03):0.034):0.12):0.264,(((((Pachyhynob:0.055,((Hynobius01:
0.038,(Hynobius_a:0.02,Hynobius00:0.019):0.01):0.011,(Ranodon_si:0.043,((Salamandre:0.042,Pseudohyno:0.065):0.008,Batra
chupe:0.041):0.006):0.006):0.01):0.027,(Onychodact:0.084,Andrias_da:0.2):0.029):0.053,((Rhyacotrit:0.22,((Eurycea_bi:0.095,(N
ototriton:0.121,Batrachose:0.112):0.02):0.015,(Plethodon_:0.099,(Phaeognath:0.052,Desmognath:0.11):0.015,(Ensatina_e:0.189
,Aneides_ha:0.085):0.015):0.017):0.059):0.036,(Lyciasalam:0.117,Ambystoma_:0.111):0.022):0.018):0.1,((Pelobates_:0.15,(((Hyl
a_japon:0.023,Hyla_chine:0.032):0.059,(Bufo_melan:0.036,Bufo_garga:0.023):0.054):0.089,(((Microhyla_:0.028,Microhyl00:0.02)
:0.044,Kaloula_pu:0.055):0.051,((Mantella_m:0.18,((Rhacophoru:0.126,Polypedate:0.201):0.052,Buergeria_:0.155):0.036):0.028,
(Limnonecte:0.245,(Rana_nigro:0.039115,Amolops_to:0.190937):0.051863):0.016348):0.071536):0.105946):0.201089):0.023047
,((Xenopus_la:0.1,Xenopus_00:0.034):0.06,(Discogloss:0.07,((Bombina_or:0.025,Bombina_00:0.012):0.086,Alytes_obs:0.09):0.0
18):0.02):0.021):0.032):0.057,((((Pangasiano:0.066,Corydoras_:0.085):0.013,(Eigenmanni:0.093,Chalceus_m:0.033):0.011):0.02
2,((Grasseicht:0.093,Chanos_cha:0.049):0.016,(Pangio_ang:0.024,((Minytrema_:0.036,Crossostom:0.068):0.007,(Schistura_:0.0
37,(Leptobotia:0.02,((Puntius_ti:0.052,Labeo_sene:0.023):0.023,(Danio_reri:0.153,(Zacco_sieb:0.018,(((Hemibarbus:0.016,Gobi
o_gobi:0.023):0.007,(Pseudaspiu:0.027,Campostoma:0.043):0.02):0.007,(Tinca_tinc:0.02,Acheilogna:0.042):0.009):0.004):0.009
):0.008):0.007):0.004):0.004):0.009):0.031):0.009):0.02,((Lates_calc:0.117,Carangoide:0.038):0.027,((Thunnus_or:0.023,Scombe
r_sc:0.062):0.034,((Plectropom:0.071,Oplegnathu:0.043,(Pagrus_maj:0.078,Lethrinus_:0.033):0.01,(Pseudolabr:0.027,Halichoer
e:0.104):0.032,Chaetodont:0.121,Antigonia_:0.067,(Percina_ma:0.025,Anarhichas:0.041):0.013,(Monodactyl:0.024,Acanthurus:0
.093):0.007,(((((Lysiosquil:0.031,(((Squilla_em:0.008,Squilla_00:0.018):0.015,Harpiosqui:0.019):0.026,Gonodactyl:0.024):0.014):
0.19,(((Macrobrach:0.221,Halocaridi:0.258):0.068,(Marsupenae:0.019,(Litopenaeu:0.016,(Penaeus_mo:0.037,Fenneropen:0.016
):0.012):0.01):0.082):0.037,((Panulirus_:0.278,Cherax_des:0.283):0.0356,(Pagurus_lo:0.17,((Geothelphu:0.226,Eriocheir_:0.136)
:0.034,(Pseudocarc:0.08,(Portunus_t:0.036,Callinecte:0.041):0.121):0.041):0.102):0.028):0.056):0.063):0.151,(Culicoides:0.298,(
(Cydistomyi:0.091,(Trichophth:0.153,((Simosyrphu:0.170,((Drosophi01:0.012,(Drosophila:0.010879,Drosophi00:0.014167):0.010
652):0.063378,(Dermatobia:0.061861,(Haematobia:0.036166,(Cochliomyi:0.013702,Chrysomya_:0.012896):0.012675):0.019):0.
05):0.011):0.015,(Ceratitis_:0.021,(Bactrocera:0.012,Bactroce00:0.023):0.02):0.051):0.039):0.023):0.054,((Anopheles_:0.019,An
ophele00:0.024):0.05,(Aedes_albo:0.048,Aedes_aegy:0.027):0.06):0.092):0.059):0.155):1.26,(((Plakortis_:0.173,Oscarella_:0.08
8):0.049,(((Halisarca_:0.038,Chondrilla:0.025):0.077,Aplysina_f:0.124):0.066,((Ephydatia_:0.067,(Topsentia_:0.079,(((Tethya_act
:0.08,((Rhabdocaly:0.116,Negombata_:0.053):0.013,Iotrochota:0.067):0.106):0.025,Geodia_nep:0.164):0.01,Axinella_c:0.089):0.
012):0.015):0.022,(((Xestospong:0.04,Callyspong:0.079):0.014,Amphimedon:0.254):0.016,Amphimed00:0.221):0.012):0.027):0.0
61):0.071,(Savalia_sa:0.074,((Nematostel:0.078,Metridium_:0.058):0.057,(Chrysopath:0.065,(Rhodactis_:0.066,(((Seriatopor:0.0
13,Pocillopor:0.007):0.104,((Montastrae:0.033,Colpophyll:0.014):0.024,Astrangia_:0.03):0.068):0.26,(Porites_po:0.04,(Siderastre
:0.027,(Agaricia_h:0.044,(Montipora_:0.014,Acropora_t:0.017):0.022):0.021):0.019):0.039):0.016):0.145):0.021):0.028):0.09):2.2
5):0.174,((Ophiura_lu:0.256,Ophiopholi:0.405):0.862,((Gymnocrinu:0.153,(Phanogenia:0.138,Florometra:0.125):0.074):0.41,(Cuc
umaria_:0.383,(((((Strongyloc:0.008,Strongyl01:0.008):0.006,Strongyl00:0.081):0.045,Paracentro:0.097):0.042,Arbacia_li:0.115):
0.18,((Pisaster_o:0.05,Asterias_a:0.062):0.085,((Luidia_qui:0.12,Astropecte:0.116):0.029,(Patiria_pe:0.076,(Acanthaste:0.022,Ac
anthas00:0.026):0.102):0.038):0.065):0.191):0.054):0.055):0.175):0.198):0.863):0.006,(Labracinus:0.164,((Neolamprol:0.039,Astr
onotus:0.071):0.016,(Cymatogast:0.083,Abudefduf_:0.029):0.006):0.015):0.019):0.006):0.004):0.034):0.127):0.068):0.031):0.064
):0.033):0.016):0.072):0.04);

nuc80 dataset

(((Acanthoscu:0.119,(Boophilus_:0.041,Ixodes_sca:0.040):0.098):0.060,(((((((Apis_melli:0.062,Nasonia_vi:0.059):0.047,((Bomby
x_mor:0.025,Spodoptera:0.020):0.118,(Diabrotica:0.057,Tribolium_:0.040):0.057):0.022):0.021,((Gryllus_bi:0.082,Locusta_mi:0.0
60):0.020,(Nilaparvat:0.104,Rhodnius_p:0.139):0.031):0.011):0.015,Pediculus_:0.132):0.048,Onychiurus:0.228):0.026,(Artemia_f
r:0.194,Daphnia_pu:0.130):0.063):0.027,(((Carcinus_m:0.057,Petrolisth:0.091):0.027,Litopenaeu:0.098):0.173,Lepeophthe:0.307
):0.027):0.037):0.038,((((((((Ambystoma_:0.040,Xenopus_tr:0.030):0.010,Homo_sapie:0.040):0.013,(Danio_reri:0.032,Tetraodon
_:0.055):0.025):0.013,Squalus_ac:0.064):0.038,(Eptatretus:0.148,Petromyzon:0.071):0.031):0.084,((Ciona_inte:0.036,Ciona_sav
i:0.040):0.110,(Halocynthi:0.132,Molgula_te:0.137):0.031):0.137):0.028,(Branchiost:0.153,Xenoturbel:0.247):0.023):0.014,((Asteri
na_p:0.126,Strongyloc:0.146):0.073,(Ptychodera:0.067,Saccogloss:0.078):0.065):0.036):0.035,(((((Aplysia_ca:0.060,(Biomphalar
:0.036,Lymnaea_st:0.033):0.035):0.097,Lottia_gig:0.223):0.032,(((Argopecten:0.107,Mytilus_ga:0.108):0.018,Crassostre:0.104):0
.025,Venerupis_:0.143):0.028):0.019,Euprymna_s:0.211):0.030,(((Capitella_:0.169,(((Helobdella:0.0685,Hirudo_med:0.124):0.05
7,Lumbricus_:0.118):0.020,Tubifex_tu:0.107):0.055):0.021,Platynerei:0.155):0.015,Chaetopter:0.152):0.017):0.030):0.022,(((((((
Ancylostom:0.024,Haemonchus:0.030):0.048,Heterorhab:0.059):0.026,(Caenorha00:0.021,Caenorhabd:0.017):0.129):0.073,Prist
ionch:0.154):0.053,(Ascaris_su:0.065,(Brugia_mal:0.018,Onchocerca:0.026):0.074):0.0607):0.028,((Bursaphele:0.192,(((Globode
ra_:0.050,Heterodera:0.051):0.059,Radopholus:0.073):0.039,Meloidogyn:0.136):0.124):0.0378,Strongyl00:0.305):0.037):0.170,Tr
ichinell:0.342):0.113,(((Dugesia_ry:0.044,Schmidtea_:0.062):0.325,((Echinococc:0.022,Taenia_sol:0.022):0.221,((Fasciola_h:0.0
94,Opisthorch:0.066):0.056,(Schistos00:0.036,Schistosom:0.035):0.094):0.094):0.122):0.072,Macrostomu:0.323):0.094):0.047);

mt68 dataset

(Adoxophyes:0.270,Trachypach:0.146,((Triops_lon:0.299,Penaeus_mo:0.216):0.047,((Scutigera_:0.326,Limulus_po:0.353):0.071
,(Priapulus_:0.421,((Metaperipa:0.326,Epiperipat:0.337):0.204,(((Platynerei:0.370,((Sipunculus:0.340,Pista_cris:0.291):0.028,(Ur
echis_ca:0.352,Lumbricus_:0.377):0.035):0.048):0.220,Loxocorone:0.598):0.137,(((Ophiura_al:0.666,(Gymnocrinu:0.371,((Stron
gyloc:0.156,Pisaster_o:0.198):0.043,Cucumaria_:0.215):0.046):0.060):0.130,(Saccogloss:0.212,Balanoglos:0.137):0.098):0.090,(
((Petromyzon:0.318,(Lepidosire:0.162,(Squalus_ac:0.110,Latimeria_:0.104):0.046):0.059):0.123,(Branchiost:0.087,Asymmetron:
0.080):0.39):0.073,((Monosiga_b:0.367,Capsaspora:0.411):0.162,((Savalia_sa:0.049,((Nematostel:0.067,Metridium_:0.047):0.04
5,(Chrysopath:0.053,(((Porites_po:0.038,(Siderastre:0.022,(Pavona_cla:0.036,Montipora_:0.034):0.012):0.009):0.032,(Ricordea_
f:0.034,Discosoma_:0.032):0.036):0.024,((Pocillopor:0.012,Madracis_m:0.020):0.072,((Montastrae:0.023,Colpophyll:0.011):0.017
,Astrangia_:0.025):0.054):0.197):0.093):0.019):0.024):0.103,((Plakortis_:0.1438,Oscarella_:0.065):0.034,((Igernella_:0.381,((Hali
sarca_:0.0302,Chondrilla:0.022):0.063,Aplysina_f:0.098):0.032):0.017,(((Xestospong:0.035,(Callyspong:0.058,Amphimedon:0.26
5):0.008):0.011,Amphimed00:0.107):0.011,(Ephydatia_:0.057,(((Suberites_:0.122,(Tethya_act:0.062,((Rhabdocaly:0.090,Negom
bata_:0.046):0.011,Iotrochota:0.051):0.076):0.011):0.021,((Topsentia_:0.052,(Ptilocauli:0.041,Ectyoplasi:0.052):0.087):0.014,(Ge
odia_nep:0.044,Cinachyrel:0.039):0.099):0.008):0.005,(Axinella_c:0.024,Agelas_sch:0.063):0.043):0.013):0.020):0.020):0.054):0.
023):0.079):1.193):0.022):0.550):0.150):0.050):0.071):0.056):0.100);

Table S5: Mean of the number of sites with a PIPn value equal to 0 according to the number of points extracted after the burn in. Test done with the nuc80 dataset.

# points	# sites with PIPn=0
100	1012
200	710
500	450
1000	324
2000	243
3000	193
5000	144

Table S6: Alignment size after removal of the most heterogeneous positions

		removed sites		alignment
		no	%	size
PIPn=0		168	8.7	1759
-ln(PIPn)	>12	165	8.6	1594
	>8	177	9.2	1417
	>6	177	9.2	1240
	>4,5	201	10.4	1039

Table S7: Posterior Probabilities (PP) of various nodes for the mtp336 dataset and 10 simulated datasets after recoding of the sequences by the 20 most frequent profiles. In bold, PP greater or equal to 0.7

model	CAT+Γ_4										
data	Real	0	1	2	3	4	5	6	7	8	9
Bilateria	**0.99**	0.02	0.53	0.03	0.46	0.01	0.02	0.12	0.04	0.18	0.03
Pancrustacea	0.69	0	0.54	0.07	0.13	0.03	0.29	0.04	0	0.25	0.12
Deuterostomia	0.23	0	0.04	0	0.02	0	0	0.04	0	0	0
Vertebrata	**0.7**	0	0.01	0	0	0	0	0.01	0	0	0
Tetrapoda	0	0	0	0	0	0	0	0	0	0	0
Amphibia	0.1	0	0.14	0.04	0.29	0.07	0.02	0	0.03	0	0
Amniota	0	0	0	0	0	0	0	0	0	0	0
Sauria	0.02	0	0	0	0	0	0	0	0	0	0
Archosauria	0.49	0	0.03	0.06	0.05	0.04	0.01	0.15	0.04	0.07	0.06
Lepidosauria	0.19	0.18	0	0.05	0	0	0	0.23	0	0.1	0.04
Mammalia	**0.84**	0	0	0	0	0	0	0.01	0	0.02	0.02

model	GTR+Γ_4										
data	Real	0	1	2	3	4	5	6	7	8	9
Bilateria	**1**	0.01	0.67	0	0.52	0	0	0.05	0.05	0.46	0.16
Pancrustacea	**1**	0.01	**0.93**	0.09	0.08	0	0.33	0.01	0	0.57	0.04
Deuterostomia	0	0	0.035	0	0.01	0	0	0	0.01	0	0
Vertebrata	**1**	0	0	0	0	0	0	0	0	0	0
Tetrapoda	**0.83**	0	0	0	0	0	0	0	0	0	0
Amphibia	**0.94**	0	0.45	0.03	0.04	0.19	0.04	0	0.01	0	0
Amniota	0.14	0	0	0	0	0	0	0	0	0	0
Sauria	0.62	0	0	0	0	0	0	0	0	0	0
Archosauria	**0.97**	0	0.01	0.06	0.02	0	0	0.12	0.06	0.1	0.05
Lepidosauria	**0.94**	0.15	0	0.01	0	0	0	0.32	0	0.14	0.05
Mammalia	**1**	0	0.04	0	0	0	0	0	0	0.02	0.02

Table S8: p-values of Khi-square tests on the distribution of profiles according to clades and physico-chemical properties of profiles. Five profile categories are considered: small, charged, aromatic, aliphatic and other properties. For mt368 and nuc80 datasets, tests were performed for all sites, most heteropecillous sites and most homopecillous sites.

Mitochondrial data	all sites	PIPn=0	PIPn~1	Site number*
all sites (1851)	0	0	$2,0e^{-102}$	480 (>=$5,7e^{-8}$)
500 fastest evolving sites (>=52,79 substitutions per site)	$2,0e^{-249}$	$6,9e^{-16}$	$1,3e^{-06}$	103 (>=$1,5e^{-5}$)

Nuclear data	all sites	PIPn=0	PIPn~1	Site number*
all sites (12608)	$7,7e^{-48}$	0,980	1	327 (>=0,595)
3000 fastest evolving sites (>=20,12 substitutions per site)	$7,0e^{-04}$	0,993	1	269 (>=0,366)
500 fastest evolving sites (>=45,77 substitutions per site)	0,996	1	1	81 (>=0,039)

*: number of sites involved in calculation for heteropecillous (i.e. PIPn=0) or homopecillous (i.e. PIPn~1). In parenthesis, smaller value of PIPn to consider homogeneity.

Table S9: Statistical supports for nodes grouping Eumetazoa and Cnidaria+Porifera according to various models of evolution. Support values are Posterior Probabilities for CAT model, and Bootstrap values for GTR and mtREV models. All inferences are conducted with 4 gamma categories.

Model (program)	Monophyletic taxa	Sequence length 1927	1759	1594	1417	1240	1039
$CAT+\Gamma_4$ (Phylobayes)	Eumetazoa	0,295	0,435	0,98	0,955	0,965	0,605
	Cnid+Pori	0,695	0,555	0,01	0,035	0,015	0,365
$GTR+\Gamma_4$ (RAxML)	Eumetazoa	0	0	2	3	7	2
	Cnid+Pori	100	100	98	97	93	98
$mtREV+\Gamma_4$ (RAxML)	Eumetazoa	0	0	0	0	1	1
	Cnid+Pori	100	100	100	100	99	97

Table S10: Evaluation of GTR+Γ4 and mtREV+Γ4 models fit by cross-validation compared with the CAT+Γ4 model on the mt68 complete dataset and subsets alignments after removal of heteropecillous sites (columns 1 and 3). In columns 2 and 4, score divided by the sequence length.

	CAT+G4 / GTR+G4		CAT+G4 / MTREV+G4	
sequence length	score likelihood fit	score / length	score likelihood fit	score / length
1927	70.7 ± 57.7	0,0367	-22.8 ± 53.9	-0,0119
1759	4.4 ± 21.6	0,0025	-83.7 ± 30.9	-0,0476
1594	-40.7 ± 31.4	-0,0255	-121.9 ± 33.0	-0,0765
1417	-52.1 ± 35.0	-0,0368	-117.2 ± 31.8	-0,0827
1240	-45.2 ± 47.0	-0,0365	-101.4 ± 47.1	-0,0818
1039	-47.5 ± 12.4	-0,0457	-99.6 ± 20.0	-0,0958
